# Incidence of community-acquired pneumonia among adults between 2016 and 2023: an observational cohort study

**DOI:** 10.1017/S0950268825100897

**Published:** 2026-01-06

**Authors:** Biying Wang, Tao Zhang, Liping Yi, Yanan Wu, Hongjie Yu, Xiaohua Liu, Youyi Zhang, Yonggen Jiang, Genming Zhao

**Affiliations:** 1 Shanghai Institute of Infectious Disease and Biosecurity, Fudan University, China; 2 Department of Epidemiology, School of Public Health, Fudan University, China; 3 Key Laboratory of Public Health Safety, Ministry of Education, China; 4 Shanghai Jiading District Center for Disease Control and Prevention, China; 5 Shanghai Minhang District Center for Disease Control and Prevention, China; 6 Shanghai Songjiang District Center for Disease Control and Prevention, China

**Keywords:** community-acquired pneumonia, COVID-19, disease burden, incidence, non-pharmacological intervention, risk factor

## Abstract

Community-acquired pneumonia (CAP) remains an important public-health problem, and the COVID-19 pandemic and non-pharmaceutical interventions (NPIs) may have altered its burden. This study aimed to provide updated CAP burden among adults in Shanghai from 2016–2023.We analysed 61,230 participants aged 20–74 years from the Shanghai Suburban Adult Cohort and Biobank. CAP episodes were ascertained via ICD codes and clinical diagnoses. We calculated incidence rates before, during, and after NPIs, conducted subgroup analyses by age, sex, comorbidity and lifestyle. We used Poisson regression to compare stages, and Cox models to identify risk factors. The Overall CAP incidence was 42.1 per 1,000 person–years (95% CI 41.3–42.8). Incidence declined during NPIs (24.2/1,000 py) and rose after NPIs (95.9/1,000 py). The inpatient-to-outpatient ratio increased to 10.1% during NPIs and fell to 5.7% post–NPI. Among those without underlying conditions, rates were 40.1, 20.1 and 73.6/1,000 py before, during and after NPIs. Incidence was higher in participants ≥60 years and in those with multiple comorbidities, especially respiratory diseases. CAP burden temporarily fell during NPIs but resurged post–NPI, notably among high–risk groups. These findings highlight the need for targeted preventive strategies and continued CAP surveillance in the post-pandemic era.

## Introduction

Community-acquired pneumonia (CAP), a prevalent and life-threatening infectious disease, is associated with approximately 3 million deaths annually [1]. High mortality and recurrence rate in hospitalized CAP patients remains a substantial public health risk. In addition, mortality rates reaching up to 50% can be observed in patients with severe complications including sepsis, respiratory failure, and acute respiratory distress syndrome (ARDS) [2]. Individuals over 65 are particularly susceptible to CAP due to higher chronic disease prevalence, necessitating targeted disease burden research for effective policy-making and management across different demographics [3, 4].

The coronavirus disease 2019 (COVID-19) pandemic, caused by severe acute respiratory syndrome coronavirus 2 (SARS-CoV-2), has significantly affected CAP disease burden, changing its etiological profile with increased incidences of viral infections [5]. Besides, evolving non-pharmacological interventions (NPIs) and vaccination strategies against rapid progression of COVID-19 pandemic necessitated long-term surveillance or frequent assessment of dynamically changed CAP burden [6]. However, currently available research for adult CAP burden during this special period in China are predominantly hospital-based and focused on etiologic alterations, particularly the varied clinical manifestations caused by SARS-CoV-2 and other respiratory pathogens [7, 8]. This underscores the need for effective longitudinal studies to capture CAP trends throughout the pandemic.

Updated population-based CAP disease burden studies covering important stages associated with NPIs are fundamental to subsequent advanced research, such as intervention evaluations. Therefore, this study aims to describe alterations in CAP disease burden including both outpatient and inpatient cases based on a prospective cohort in Shanghai, depicting CAP incidences before, during, and after NPIs. In addition, the ratio of inpatient-to-outpatient (IOR) CAP incidence and population variations in CAP disease burden were analysed for all three periods, providing policymakers with evidence for more tailored strategies and efficient healthcare resource allocation.

## Methods

### Study design and population

The study was conducted based on Shanghai Suburban Adult Cohort and Biobank (SSACB). SSACB is a prospective cohort that recruited 69,116 individuals aged 20–74 years from four districts (Songjiang, Jiading, Xuhui, and Minhang) in Shanghai between April 2016 and December 2019 [9, 10]. Upon enrolment, subjects completed electronic questionnaires administered by trained interviewers to gather baseline characteristics like demographic and epidemiological data. They also underwent physical exams for height, weight, blood pressure, and heart rate and provided biochemical test data. The cohort follow-ups involved in-person surveys and annual queries of health information system [9, 10]. The health information system encompasses all medical records from outpatient and inpatient visits across various levels of hospitals throughout the entire Shanghai city.

### Covariates and data collection

Data on demographic characteristics, chronic diseases from baseline surveys were included in analyses [11]. Underlying diseases were determined based on self-reported history and physical examination results. Smoking was defined as smoking at least one cigarette daily for a continuous period of 6 months or more, and it was classified into never smokers and smokers. Drinking was defined as drinking alcohol at least three times weekly for 6 months or more. Body mass index (BMI) was calculated determined by dividing weight in kilograms by the square of their height in meters. BMI was categorized using criteria recommended for Chinese populations: underweight (<18.5 kg/m^2^), normal weight (18.5–24 kg/m^2^), overweight (24–28 kg/m^2^), and obese (≥28 kg/m^2^).

### Identification of CAP cases

Medical visit records of the enrolled subjects, including Outpatient and hospitalization, were queried from health information system by personal identification codes. The CAP episodes were identified by ICD-10 codes (J12–J18) or term ‘pneumonia’, excluding ‘chemical pneumonia’ and other non-relevant cases. Multiple visits by the same patient within 30 days were counted as a single CAP episode [11]. Outpatient records overlapped with hospitalization were excluded.

### Statistical analysis

Valid time period was set between the start of SSACB enrolment (June 6, 2016) and the time of last clinical record (31 March, 2023). Outpatient diagnosis dates and hospital admission dates were indexed for CAP cases. The date of baseline survey for each individual was recorded as the start date of follow-up. When taking full CAP episodes as the outcome, the observation endpoints were marked as the date of death or end of cohort observation. First-time CAP occurrences were also considered as endpoints when focusing on the primary cases in sensitivity analysis.

The overall observation period was split into three stages according to the onset and cessation of NPIs against SARS-CoV-2 in Shanghai: (1) Before NPIs: June 6, 2016 to February 9, 2020; (2) During NPIs: February 10, 2020 to December 9, 2022; (3) After NPIs: December 10, 2022-March 31, 2023. The implementation of NPIs was marked by the municipal notice ‘*Shanghai Municipal Government Notice on Further Strengthening the Implementation of COVID-19 Prevention and Control Measures’*, cessation of NPIs was marked by the notice ‘*Lifting of Control in 38 High-Risk Areas in Shanghai’.*

Overall, outpatient, and inpatient CAP incidences were calculated and Poisson regression was performed to estimate 95% CI and incidence rate ratios (IRRs). Annual CAP incidences adjusted with age, sex, and stratified by district were also summarized using age- and sex-specific population from Shanghai Population Census Yearbook 2020, with bootstrap sampling of 1,000 times calculating 95% CI [12]. We also compared incidences among subgroups based on Poisson regression. Incidence rates of both outpatient and hospitalized CAP cases were also assessed separately for different stages NPIs stages. The IOR was defined as the inpatient incidence divided by the outpatient incidence within the same phase and stratum. CAP incidences among subpopulation were also estimated considering demographic characteristics, lifestyles, and underlying conditions. Variations in CAP incidence between observation stages were analysed using Poisson regression. Risk factors for CAP were estimated using Cox regression models with shared Weibull frailty. We further adjusted for socioeconomic status, lifestyle, and underlying condition variables to control their potential contributions. To minimize bias from hospital-acquired pneumonia, we excluded subjects with hospital admissions in the prior 7 days or pneumonia symptoms developing after hospitalization [13]. All analyses were performed using R, version 4.2. A two-tailed *P*-value <0.05 was considered significant.

### Ethics approval

This study was approved by the Medical Research Ethics Committee of the School of Public Health, Fudan University (IRB approval number 2016-2104-0586-S1), and all participants provided written informed consent.

## Results

### Study population and characteristics

**Figure 1.**
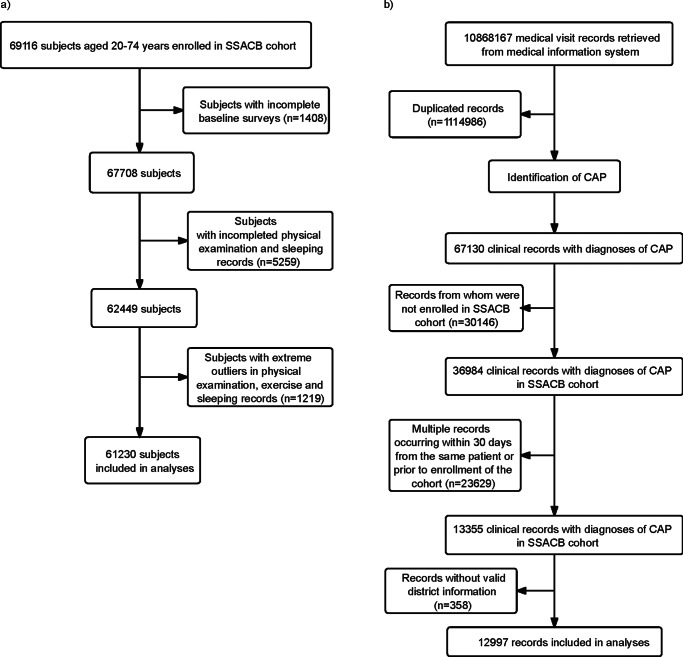
Flow chart of inclusion and exclusion of the study cohort members and CAP cases. (a) Flow chart of inclusion and exclusion of the study cohort members. (b) Flow chart of identification of the CAP cases.

SSACB had recruited 69,116 subjects by December 2019, with 97.1% (67,708) having completed baseline surveys. After excluding subjects with incomplete responses, physical examination, and extreme outliers, 61,230 persons, with over 309,121 person-years, were included (Figure 1a). A total of 10,868,167 medical records of all subjects were retrieved from the health information system dated till March 31, 2023. After removal of duplicated ones, 9,753,181 records remained and 67,130 were diagnosed with CAP. After removing the CAP records before enrolment dates, a total of 12,997 CAP records were selected for the analysis (Figure 1b).

Characteristics including socio-demographic data, personal lifestyles, and underlying diseases were summarized by age strata in Table 1. The median age at baseline was 59 years old [interquartile range (IQR): 14.82], with females making up 59.6% of subjects, a trend consistent across age groups. Subjects aged over 60 years mostly had received primary-school education while those under 60 often possessed middle-school diplomas. Subjects over 50 were more prone to be obese or overweight. Less than one third (28.0%) of the overall baseline population were free from underlying chronic diseases, with disease prevalence increasing with age. Majority of those with comorbidities were presented with one chronic disease (47.0%), followed by two types simultaneously (33.8%), three and more (19.2%). During total follow-up period, 57.5% (34,512/60,040) persons received three-dose COVID-19 vaccines, and 25.1% (15,050/60,040) not vaccinated.

**Table 1.** Distribution of baseline characteristics by age

Characteristics	20–50 years (N = 14,268)	51–60 years (N = 19,282)	61–70 years (N = 23,165)	>70 years (N = 4,515)	Overall (N = 61,230)
**Sex, n (%)**					
Male	5,478 (38.4)	6,866 (35.6)	10,233 (44.2)	2,158 (47.8)	24,735 (40.4)
Female	8,790 (61.6)	12,416 (64.4)	12,932 (55.8)	2,357 (52.2)	36,495 (59.6)
**Education level, n (%)**					
No formal school	201 (1.4)	1,122 (5.8)	3,887 (16.8)	923 (20.4)	6,133 (10.0)
Primary school	1,521 (10.7)	3,940 (20.4)	8,514 (36.8)	1,422 (31.5)	15,397 (25.1)
Middle school	5,349 (37.5)	9,650 (50.0)	7,036 (30.4)	1,217 (27.0)	23,252 (38.0)
High school or above	7,197 (50.4)	4,570 (23.7)	3,728 (16.1)	953 (21.1)	16,448 (26.9)
**Retired, n (%)**					
**Retired, n (%)**	1,113 (7.8)	11,311 (58.7)	22,047 (95.2)	4,305 (95.3)	38,776 (63.3)
**Medical insurance, n (%)**					
Uninsured	68 (0.5)	82 (0.4)	84 (0.4)	26 (0.6)	260 (0.4)
UEBMI	7,684 (53.9)	6,612 (34.3)	4,962 (21.4)	885 (19.6)	20,143 (32.9)
URBMI, NRCMI, or URCMI	6,160 (43.2)	12,215 (63.3)	17,711 (76.5)	3,484 (77.2)	39,570 (64.6)
Other types of insurance	356 (2.5)	373 (1.9)	408 (1.8)	120 (2.7)	1,257 (2.1)
**BMI group, n (%)**					
Underweight	712 (5.0)	422 (2.2)	512 (2.2)	109 (2.4)	1755 (2.9)
Normal	7,578 (53.1)	8,865 (46.0)	9,926 (42.8)	1920 (42.5)	28,289 (46.2)
Overweight	4,528 (31.7)	7,698 (39.9)	9,650 (41.7)	1807 (40.0)	23,683 (38.7)
Obese	1,450 (10.2)	2,297 (11.9)	3,077 (13.3)	679 (15.0)	7,503 (12.3)
**Smoking, n (%)**					
Non-smoker	11,975 (83.9)	15,486 (80.3)	18,840 (81.3)	3,869 (85.7)	50,170 (81.9)
Smoker	2,293 (16.1)	3,796 (19.7)	4,325 (18.7)	646 (14.3)	11,060 (18.1)
**Drinking, n (%)**					
Non-drinker	13,227 (92.7)	17,273 (89.6)	20,221 (87.3)	4,004 (88.7)	54,725 (89.4)
Drinker	1,041 (7.3)	2009 (10.4)	2,944 (12.7)	511 (11.3)	6,505 (10.6)
**Underlying diseases**					
Cardiovascular and cerebrovascular diseases, n (%)	3,254 (22.8)	10,041 (52.1)	15,796 (68.2)	3,495 (77.4)	32,587 (53.2)
Respiratory diseases, n (%)	762 (5.3)	1,572 (8.2)	2,561 (11.1)	625 (13.8)	5,520 (9.0)
Diabetes, n (%)	751 (5.3)	3,075 (15.9)	5,009 (21.6)	1,096 (24.3)	9,931 (16.2)
Cancer, n (%)	84 (0.6)	341 (1.8)	618 (2.7)	140 (3.1)	1,183 (1.9)
Other diseases, n (%)	4,198 (29.4)	7,765 (40.3)	9,875 (42.6)	1828 (40.4)	23,664 (38.6)
**Comorbidity, n (%)**					
None	7,712 (54.1)	5,133 (26.6)	3,766 (16.3)	511 (11.3)	17,122 (28.0)
One	4,345 (30.5)	7,047 (36.5)	7,834 (33.8)	1,461 (32.4)	20,687 (33.8)
Two	1770 (12.4)	4,884 (25.3)	6,894 (29.8)	1,405 (31.1)	14,953 (24.4)
Three and more	441 (3.1)	2,218 (11.5)	4,671 (20.2)	1,138 (25.2)	8,468 (13.8)

### Overall CAP incidence patterns by baseline characteristics

During 309,121 person-years of follow-up, 12,997 pneumonia cases were identified in 8,214 cohort members and 2,412 (29.4%) members experienced recurrent episodes. The overall incidence rate of CAP was 42.1/1000/year (95% CI: 41.3–42.8), with 39.2 (95% CI: 38.5–39.9) outpatient and 2.8 (95% CI: 2.6–3.0) inpatient cases per 1,000 person-years occurring during observation.

The overall and outpatient incidence rates were higher among female subjects, while male subjects had higher hospitalization rates (*P* < 0.001). An increased trend of incidence rates with age was observed regardless of types of CAP patient care (Table 2). Besides, CAP inpatients tended to receive relatively lower education level, smoke heavily and be underweight or obese (Table 2). Subjects with underlying conditions such as cardiovascular and cerebrovascular diseases, respiratory diseases, diabetes, cancer, or other diseases were more susceptible to CAP, particularly those with multiple chronic conditions. Similar patterns were observed in first-time CAP as well (Supplementary Table S1).

**Table 2.** Incidence of CAP by baseline characteristics

Characteristics	Incidence density of CAP episodes (/1,000 person-years)	Hospitalization density of CAP episodes (/1,000 person-years)	Outpatient density of CAP episodes (/1,000 person-years)
**Sex**			
Male	38.0 (36.9–39.1)	3.3 (3.0–3.7)	34.7 (33.6–35.7)
Female	44.8 (43.9–45.8)	2.5 (2.2–2.7)	42.4 (41.4–43.3)
**Age at baseline**			
20–50 years	28.4 (27.2–29.7)	0.8 (0.6–1.0)	27.7 (26.5–28.9)
51–60 years	43.6 (42.3–44.9)	1.9 (1.6–2.1)	41.7 (40.5–43.0)
61–70 years	46.7 (45.4–47.9)	4.0 (3.6–4.4)	42.7 (41.5–43.9)
> 70 years	58.8 (55.6–62.1)	8.4 (7.2–9.7)	50.4 (47.4–53.5)
**Education level**			
No formal school	52.3 (49.8–54.8)	5.8 (5.0–6.7)	46.5 (44.2–48.9)
Primary school	52.7 (51.1–54.3)	4.2 (3.8–4.7)	48.5 (47.0–50.0)
Middle school	40.2 (39.0–41.3)	2.1 (1.9–2.4)	38.0 (36.9–39.2)
High school	29.6 (28.4–30.9)	1.2 (1.0–1.4)	28.5 (27.3–29.7)
**Medical insurance**			
Uninsured	42.6 (32.9–54.1)	3.4 (1.2–7.4)	39.2 (29.9–50.2)
UEBMI	37.9 (36.7–39.1)	1.5 (1.3–1.8)	36.4 (35.2–37.6)
URBMI, RCMI, or URCMI	44.4 (43.5–45.3)	3.5 (3.2–3.7)	40.9 (40.1–41.8)
Other types of insurance	32.2 (28.0–36.7)	2.5 (1.5–3.9)	29.7 (25.7–34.1)
**BMI group**			
Underweight	47.1 (42.7–51.8)	4.5 (3.2–6.0)	42.6 (38.4–47.1)
Normal	39.5 (38.5–40.5)	2.7 (2.4–3.0)	36.8 (35.8–37.8)
Overweight	42.3 (41.1–43.5)	2.7 (2.4–3.0)	39.6 (38.5–40.7)
Obese	49.8 (47.6–52.1)	3.3 (2.7–3.9)	46.6 (44.4–48.8)
**Smoking**			
Non-smoker	43.9 (43.1–44.7)	2.8 (2.6–3.0)	41.1 (40.3–41.9)
Smoker	34.1 (32.7–35.7)	3.0 (2.6–3.5)	31.1 (29.7–32.6)
**Drinking**			
Non-drinker	42.3 (41.5–43.0)	2.7 (2.5–2.9)	39.5 (38.8–40.3)
Drinker	40.4 (38.3–42.6)	3.5 (2.9–4.1)	36.9 (34.9–39.0)
**Underlying conditions**			
Cardiovascular and cerebrovascular diseases	45.6 (44.6–46.7)	3.4 (3.2–3.7)	42.2 (41.2–43.2)
Respiratory diseases	93.7 (90.0–97.4)	7.2 (6.2–8.3)	86.5 (82.9–90.1)
Diabetes	48.3 (46.3–50.3)	3.9 (3.4–4.5)	44.3 (42.5–46.2)
Cancer	53.4 (47.4–59.8)	6.2 (4.3–8.5)	47.2 (41.6–53.3)
Other diseases	43.1 (41.9–44.3)	2.9 (2.6–3.3)	40.2 (39.1–41.4)
**Comorbidity**			
None	33.1 (32.0–34.3)	1.6 (1.4–1.9)	31.5 (30.4–32.7)
One	39.6 (38.5–40.9)	2.5 (2.2–2.8)	37.2 (36.0–38.4)
Two	45.8 (44.2–47.3)	3.5 (3.1–4.0)	42.2 (40.8–43.7)
Three and more	61.9 (59.4–64.3)	5.2 (4.5–5.9)	56.7 (54.4–59.1)

### CAP incidence and IOR of different time periods

**Figure 2.**
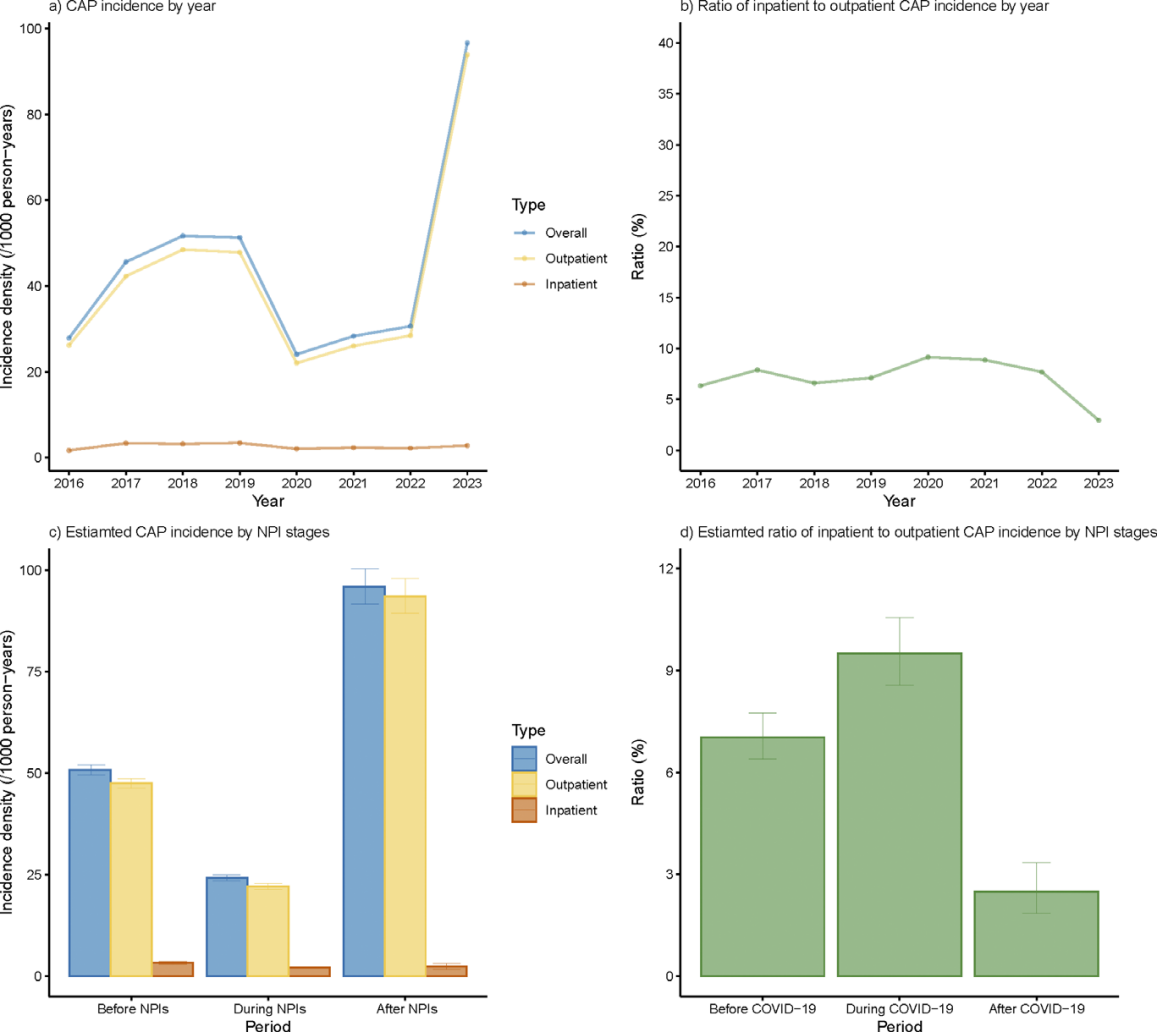
Annual incidence rate of overall, outpatient, and inpatient CAP cases under different NPI stages. (a) Annual CAP incidence density under different NPI stages. (b) Annual ratio of inpatient to outpatient CAP incidence under different NPI stages. (c) Estimated CAP incidence by different NPI stages. (d) Estimated ratio of inpatient to outpatient CAP incidence by different NPI stages.

The overall incidences of CAP before, during, and after NPIs were 50.8, 24.2, and 95.9/1000/year separately (Figure 2c). After adjusted with age, sex, and stratified by district, the overall and outpatient incidence densities underwent a sharp decline in 2020 and increased steeply in 2023, surpassing the level before NPIs (Supplementary Figures S1a and S1c). The adjusted inpatient CAP incidence also showed an increase in 2023, though it was less pronounced compared to the rise observed in outpatient incidence. Both overall and outpatient incidence densities rose in 2021, yet they failed to rebound to the level before NPIs during the whole NPI period but doubled after the cessation of NPIs.

Though both CAP outpatient and inpatient incidences experienced a precipitous drop during NPIs, a higher proportion of CAP patients tend to receive inpatient treatment during NPIs in general, leading to elevated IOR (Figure 2b,d). After cessation of NPIs, a significant decline was observed in IOR, which was also much lower than that before NPIs (Figure 2b,d).

### CAP incidence among subpopulations

Similar trend of decreased and increased CAP incidence during and after NPIs was observed among subgroups related to demographic, life-style, and underlying diseases (*P* < 0.001) (Table 3). Females had a higher CAP incidence both before and after NPIs but not during the pandemic (Table 3). However, elevated inpatient incidence was observed in males across all three periods (Supplementary Table S2). Consistent with the predisposition on estimated CAP burden throughout the observation period, CAP incidences were higher among older individuals and those with lower educational levels in all three periods regardless of type of admission (Table 3, Supplementary Tables S2 and S3). Notably, CAP incidence among individuals aged 60 and older after NPIs were twice as high as prior to NPIs, and specifically three times higher among those aged 70 and above.

**Table 3.** CAP incidence across various NPI stages among subpopulations

Characteristics	CAP incidence before NPIs (/1,000 person-years)	CAP incidence during NPIs (/1,000 person-years)	CAP incidence after NPIs (/1,000 person-years)	IRR for During vs. Before	P value for During vs. Before	IRR for After vs. During	P value for After vs. During
**Sex**							
Male	43.2 (41.4–44.9)	26.0 (24.7–27.3)	91.9 (85.0–99.3)	0.60 (0.56–0.64)	< 0.001	3.54 (3.23–3.88)	< 0.001
Female	56.0 (54.4–57.7)	25.3 (24.3–26.3)	105.3 (99.3–111.6)	0.45 (0.43–0.47)	< 0.001	4.17 (3.89–4.47)	< 0.001
**Age at baseline**							
20–50 years	36.3 (34.3–38.4)	18.0 (16.6–19.3)	57.0 (50.0–64.6)	0.49 (0.45–0.54)	< 0.001	3.17 (2.74–3.68)	< 0.001
51–60 years	54.2 (52.1–56.4)	24.6 (23.3–25.9)	89.3 (81.9–97.1)	0.45 (0.42–0.48)	< 0.001	3.63 (3.28–4.02)	< 0.001
61–70 years	55.4 (53.3–57.6)	28.5 (27.2–29.9)	124.7 (116.2–133.5)	0.51 (0.48–0.55)	< 0.001	4.37 (4.02–4.76)	< 0.001
> 70 years	63.3 (58.2–68.8)	40.0 (36.4–43.8)	168.8 (146.8–192.9)	0.63 (0.56–0.71)	< 0.001	4.22 (3.58–4.98)	< 0.001
**Education level**							
No formal school	59.9 (56.2–63.9)	31.4 (28.8–34.1)	112.0 (97.6–127.9)	0.52 (0.47–0.58)	< 0.001	3.57 (3.04–4.18)	< 0.001
Primary school	59.0 (56.6–61.4)	32.8 (31.1–34.5)	115.1 (105.8–125.0)	0.56 (0.52–0.59)	< 0.001	3.52 (3.19–3.88)	< 0.001
Middle school	49.0 (47.1–50.9)	23.5 (22.3–24.7)	92.8 (85.8–100.2)	0.48 (0.45–0.51)	< 0.001	3.95 (3.60–4.33)	< 0.001
High school	38.6 (36.4–40.9)	18.0 (16.7–19.4)	89.4 (80.6–98.9)	0.47 (0.42–0.51)	< 0.001	4.96 (4.37–5.62)	< 0.001
**Medical insurance**							
Uninsured	53.7 (38.7–72.1)	14.6 (7.6–25.0)	138.6 (72.0–237.4)	0.27 (0.14–0.53)	< 0.001	9.54 (4.14–22.01)	< 0.001
UEBMI	47.9 (45.8–50.0)	22.8 (21.5–24.1)	95.0 (87.3–103.3)	0.47 (0.44–0.51)	< 0.001	4.18 (3.77–4.62)	< 0.001
URBMI, NRCMI, or URCMI	52.7 (51.2–54.2)	27.0 (26.0–28.0)	103.0 (97.3–109.0)	0.51 (0.49–0.54)	< 0.001	3.82 (3.57–4.08)	< 0.001
Other types of insurance	32.1 (25.9–39.2)	25.8 (20.7–31.7)	77.8 (52.4–110.3)	0.80 (0.60–1.08)	0.15	3.01 (1.97–4.62)	< 0.001
**BMI group**							
Underweight	59.4 (52.0–67.4)	32.5 (27.6–38.1)	54.2 (36.2–77.2)	0.55 (0.44–0.67)	< 0.001	1.67 (1.11–2.51)	< 0.05
Normal	47.7 (46.0–49.4)	24.5 (23.4–25.7)	94.5 (88.0–101.4)	0.51 (0.48–0.54)	< 0.001	3.86 (3.54–4.20)	< 0.001
Overweight	50.9 (49.0–52.9)	25.4 (24.1–26.6)	102.3 (94.9–110.0)	0.50 (0.47–0.53)	< 0.001	4.03 (3.69–4.41)	< 0.001
Obese	60.2 (56.5–64.0)	28.4 (26.1–30.7)	124.0 (109.9–139.3)	0.47 (0.42–0.52)	< 0.001	4.37 (3.79–5.05)	< 0.001
**Smoking**							
Non-smoker	53.9 (52.5–55.3)	24.4 (23.6–25.2)	101.9 (97.0–106.9)	0.45 (0.43–0.47)	< 0.001	4.17 (3.94–4.43)	< 0.001
Smoker	38.4 (36.1–40.8)	23.2 (21.8–24.7)	68.2 (59.8–77.3)	0.60 (0.55–0.66)	< 0.001	2.94 (2.53–3.40)	< 0.001
**Drinking**							
Non-drinker	51.8 (50.5–53.1)	23.8 (23.0–24.5)	97.1 (92.5–101.8)	0.46 (0.44–0.48)	< 0.001	4.08 (3.86–4.33)	< 0.001
Drinker	43.6 (40.4–47.0)	27.8 (25.5–30.3)	86.1 (74.0–99.5)	0.64 (0.57–0.71)	< 0.001	3.10 (2.61–3.68)	< 0.001
**Underlying conditions**							
Cardiovascular and cerebrovascular diseases	54.5 (52.8–56.3)	25.9 (24.8–26.9)	112.6 (106.2–119.2)	0.47 (0.45–0.50)	< 0.001	4.36 (4.06–4.67)	< 0.001
Respiratory diseases	118.4 (112.1–124.9)	47.1 (43.9–50.4)	152.3 (135.5–170.5)	0.40 (0.36–0.43)	< 0.001	3.23 (2.83–3.70)	< 0.001
Diabetes	57.7 (54.4–61.1)	27.1 (25.2–29.0)	125.1 (113.0–137.9)	0.49 (0.46–0.52)	< 0.001	4.57 (4.18–4.98)	< 0.001
Cancer	69.0 (58.4–80.9)	27.0 (21.8–32.9)	139.2 (104.1–181.4)	0.41 (0.34–0.49)	< 0.001	5.17 (4.02–6.66)	< 0.001
Other diseases	53.1 (51.1–55.2)	24.4 (23.3–25.6)	100.2 (93.2–107.6)	0.46 (0.43–0.49)	< 0.001	4.10 (3.76–4.47)	< 0.001
**Comorbidity**							
None	40.1 (38.2–42.1)	20.1 (18.8–21.4)	73.6 (66.4–81.3)	0.50 (0.46–0.54)	< 0.001	3.66 (3.25–4.13)	< 0.001
One	48.6 (46.6–50.6)	24.0 (22.7–25.3)	89.2 (81.9–97.0)	0.49 (0.46–0.53)	< 0.001	3.72 (3.36–4.11)	< 0.001
Two	55.2 (52.6–57.8)	27.6 (25.9–29.3)	114.1 (104.2–124.6)	0.50 (0.46–0.55)	< 0.001	4.14 (3.72–4.61)	< 0.001
Three and more	75.8 (71.6–80.2)	38.1 (35.5–40.8)	162.0 (146.1–179.0)	0.50 (0.46–0.54)	< 0.001	4.25 (3.76–4.81)	< 0.001

Increased CAP incidence was observed among participants with obesity. Following the cessation of NPIs, CAP incidences among individuals who were obese or overweight were twice as high as before NPIs. In contrast, the increase among those with normal weight or underweight was relatively modest. Whereas during the NPIs, the inpatient incidence was higher in the underweight group (Table 3).

Before NPIs, there were 40.1 (95% CI: 38.2–42.1) CAP cases per 1,000 person-years among people without underlying diseases, and 2.0 (95% CI: 1.6–2.5) hospitalized cases and 38.1 (95% CI: 36.2–40.0) outpatient cases occurred correspondingly. With the increase of number of underlying conditions, CAP incidence rose correspondingly, and those with more than two types of diseases had nearly twice the incidence compared with participants free of chronic diseases. Following the cessation of NPIs, CAP incidence among individuals with more than two types of underlying diseases rose dramatically to 162.0 (95% CI: 146.1–179.0) per 1,000 person-years. In particular, respiratory diseases were notably elevated CAP incidences across all three periods (Table 3). Throughout all three periods, it was consistently found that non-smokers and non-drinkers were associated with higher CAP incidence (Table 3). As for CAP hospitalization, such tendency was also found except for during-NPI period (Supplementary Table S2).

## Discussion

To the best of our understanding, this is the first prospective population-based study estimated disease burden of CAP under the impact of COVID-19 and NPIs, providing both outpatient and inpatient CAP incidence in China. We observed elevated CAP incidence among subjects who were females, older than 70 years, with low education level, or with underlying diseases. Estimated inpatient CAP incidence of 3.3 (95% CI: 3.0–3.7) per 1,000 person-years before NPIs was in accordance with that of 3.1 (95% CI: 3.1–3.2) per 1,000 person-years based on the study in southeastern China from 2015 to 2019, adding validity to our results [14]. The pre-NPI incidence of CAP from our study was higher compared with previous report from Eastern China (50.8/1000/year vs. 12.1/1000/year) [15]. The discrepancy may attribute to differences in case definition and data sources. The latter study applied 90-day period free of CAP claim records to identify one CAP episode from another while we used the 30-day interval, which was commonly used as the threshold to distinguish between the initial episode and a new one [16]. The difference in case definitions may partially explain the differences, approximately three-fold higher incidence in our study than in the latter one. In addition, we utilized all outpatient and inpatient medical visits captured in the health information system in Shanghai while the prior study only included residents with Urban Basic Medical Insurance, leading to underestimated incidence as uninsured individuals were associated with higher CAP incidence.

The sharp decline in CAP incidence during NPIs was in consistence with the trend observed in a retrospective study in Shenzhen in 2020, although only a brief span after the onset of COVID-19 were encompassed [5]. This shift was associated with the implementation of NPIs in China – quarantines, social distancing, mask-wearing, and hand hygiene – which curtailed respiratory diseases through limiting person-to-person contact [17]. Following the cessation of NPIs, subsequent increase in CAP incidence was observed, which was also found in a study conducted in Italy [18]. Prolonged NPI measures might lead to a temporary reduction in population-level immunity to common respiratory pathogens due to decreased exposure, potentially resulting in higher susceptibility once lifted. Therefore, cessation of such interventions could contribute to a sharp rise in CAP incidence.

IOR summarizes the relative shift between hospitalized and outpatient CAP burden. IOR may reflect greater severity or reductions in outpatient capture, as seen when care-seeking and service availability changed. Beyond the overall alterations in CAP incidence rates during different stages of NPIs, IOR among observed participants also shifted. Generally, more CAP patients received hospitalized treatment during NPIs than before and after NPIs. For fear of getting infected with SARS-CoV-2, people might have been reluctant to seek for primary medical help during the pandemic, leading to decreased outpatient CAP incidence during NPIs [19, 20]. Additionally, under ‘Zero-COVID’ policy in China, symptom surveillance in healthcare facilities and communities captured patients visiting fever clinics and transferred them directly for inpatient treatment, potentially increasing IOR during NPIs [21, 22]. After the cessation of NPIs, a decreased IOR were observed along with a dramatic rise in CAP outpatient incidence. The proportion of inpatient care has often been used as an indicator of disease severity [23]. However, the decrease in IOR after NPIs did not imply reduced severity of CAP but rather suggested that the decrease was actually the result of an overburdened healthcare system unable to meet the demand for hospitalization. Those who might have avoided seeking care for CAP symptoms during NPIs resumed normal healthcare-seeking behaviours, leading to an initial surge in outpatient visits and decreased IOR. It was also worth noting that compared with the steep rise in CAP cases, the limited amount of hospital beds cannot meet the demand of every patient, thereby leading to reduced IOR.

Advanced age was associated with elevated CAP incidence and higher sensitivity to the cessation of NPIs, suggesting that individuals over 50 – especially those 70 and above – may have become more reliant on NPI measures for protection, due to both biological vulnerabilities and reduced overall resilience. Participants with obesity also exhibited abrupt increase. It was noteworthy that subjects with comorbidities, especially respiratory diseases, had higher CAP incidence during the pandemic and this trend tended to be more pronounced with number of underlying chronic diseases. Individuals with multiple comorbidities often have compromised immune systems, making them more susceptible to infectious diseases [24]. This highlights the need for tailored public health strategies for high-risk groups. Smoking was observed to be associated with a decline in both overall and outpatient CAP incidence while the inpatient cases showed opposite trend, possibly due to their poor health awareness and underutilization of medical services, leading to fewer outpatient visits and outpatient expenditures [25–28]. Based on further multivariable analyses, the excess risk linked to respiratory diseases persisted after adjustment, and the gradient with comorbidity count remained evident. The ‘non-smoker’ excess attenuated after adjustment yet did not fully disappear for CAP, suggesting residual confounding and care-seeking differences. Notably, completion of a three-dose COVID-19 vaccination series was associated with reduced CAP risk during and after the pandemic, particularly for inpatient cases. These adjusted findings support targeted prevention in older adults and those with respiratory comorbidities.

Since the emergency use of vaccine in June 2022, China initiated a COVID-19 vaccination program. It was reported in previous studies that COVID-19 vaccination played an important role in both prevention of pneumonia associated with SARS-CoV-2 and alleviating disease severity [29]. Both by the time of NPIs cessation and in the period following that, the majority received COVID-19 vaccinations (74.9% and 75.7%), predominantly opting for three-dose programs. The coverage among participants aged 60 years and younger was in accordance with the study using data on employees in China, further suggesting the representativeness and reference value of our results [30].

The main strength of our study attributes to the use of a prospective and community-based cohort, which ensures the representativeness of the general population. The long observation period of this study, which spans before, during, and after NPIs, allows for capture of the dynamic changes in CAP disease burden. We calculated both outpatient and inpatient incidence based on the health information system, which captured nearly all the episodes and enhanced the accuracy of our estimated disease burden. In addition, IOR was calculated for addressing the heavy burden of CAP. The detailed estimation of CAP incidence across various subgroups contributes to identification of high-risk populations, aiding in targeted public health interventions.

There are certain limitations in our study. First, only residents in four districts in Shanghai were included in SSACB. To address potential concerns about representativeness, we selected four districts that were geographically dispersed across Shanghai, including both urban and suburban populations, enhancing the generalizability of our findings. Second, we used the term ‘pneumonia’ to identify CAP cases for records without ICD-10 codes, which was only a small proportion of the cases. Additionally, all retrieved CAP records were checked manually to rule out irrelevant diagnoses, in order to avoid misclassification. Third, the latest medical visit record was dated till March 31, 2023, indicating that the post-NPI analysis covered a relatively short period. Although this window includes winter–spring, the peak season for CAP, which informs the immediate post-NPI impact, it may inflate annualized incidence estimates relative to longer phases. Further analysis with broader window should be considered. In addition, covariates were available only at baseline due to data limitations, and subsequent changes were not uniformly captured. This may introduce misclassification of time-varying risk factors and bias and we will further update analyses with time-updated covariates to refine estimates. Non-COVID vaccinations were not included in the analyses due to incomplete vaccination records for four districts. Future incorporation of other related vaccines will be performed with the updated immunization data. While our multivariable Cox models adjust for measured confounders and provide evidence of independent associations, unmeasured confounding and time-varying exposures may still limit causal interpretation. Future studies employing longitudinal analytic approaches, such as marginal structural models with time-updated covariates would further strengthen causal inference.

## Conclusions

In the present study, we discovered that CAP continued to pose a significant health burden, particularly among individuals >50 years and those with underlying conditions. This burden was exacerbated following the cessation of NPIs. Notably, IOR of CAP peaked during NPIs and declined after cessation, indicating a shift in disease severity and healthcare utilization patterns over these phases. The findings emphasized the urgent need for targeted prevention during epidemic outbreaks including vaccination programs aimed at susceptible populations and periods to alleviate the burden of CAP in China.

## Supporting information

10.1017/S0950268825100897.sm001 Wang et al. supplementary material Wang et al. supplementary material

## Data Availability

The datasets generated and analysed during the current study are not publicly available due to the data of SSACB not being open access but are available from the corresponding author on reasonable request.

## Supplementary material

The supplementary material for this article can be found at http://doi.org/10.1017/S0950268825100897.
