# Supplementary material for: Incidence of community-acquired pneumonia among adults between 2016 and 2023: an observational cohort study
Source: Epidemiol Infect. 2026 Jan 6;154:e15. doi: 10.1017/S0950268825100897 (PMC12835933; doi:10.1017/S0950268825100897)
Supplement: Wang et al. supplementary material [file S0950268825100897sup001.zip › FS2.pdf]

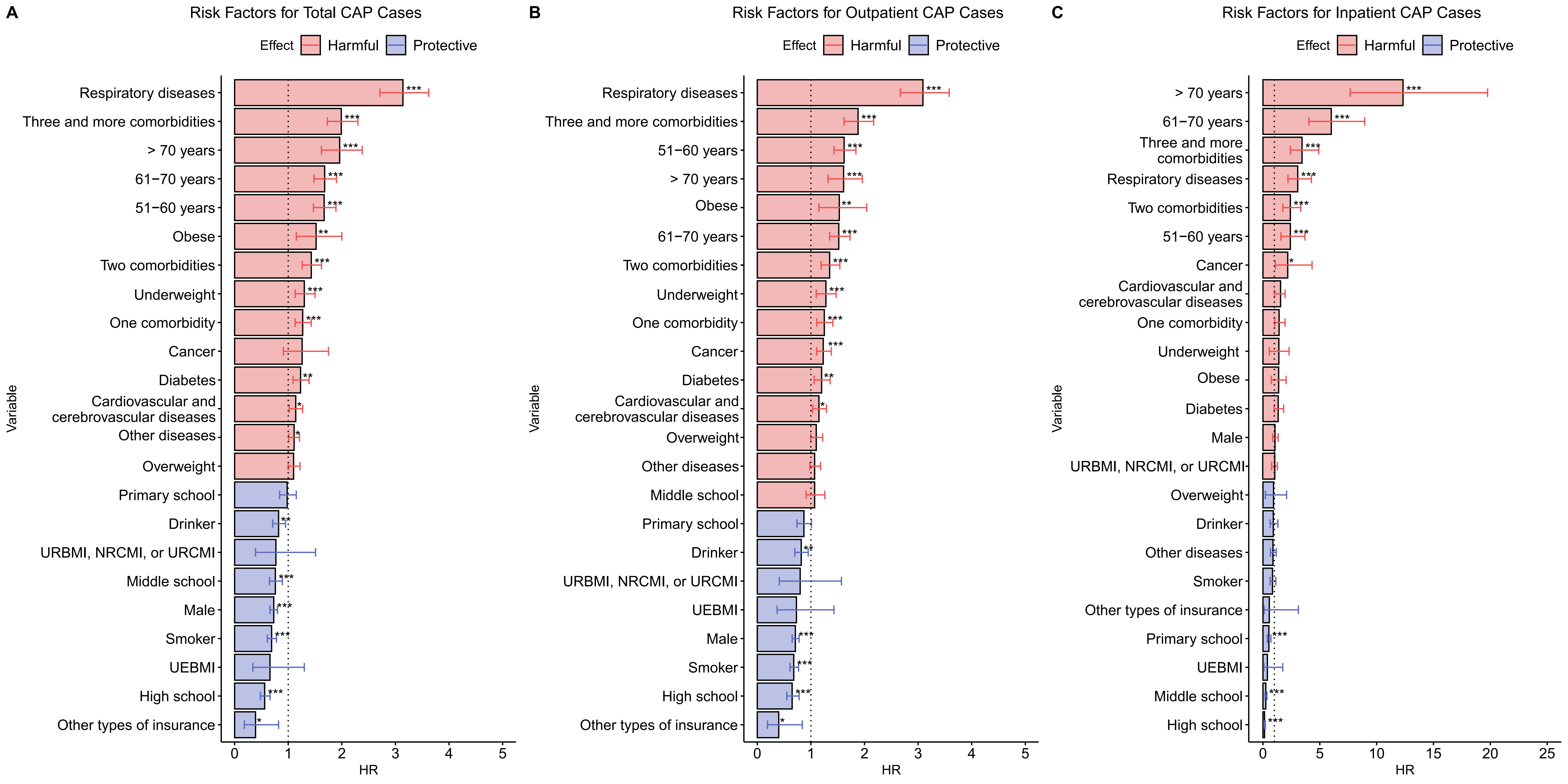
A
Risk Factors for Total CAP Cases
Effect Harmful Protective
Respiratory diseases
Three and more comorbidities
> 70 years
61−70 years
51−60 years
Obese
Two comorbidities
Underweight
One comorbidity
Cancer
Diabetes
Cardiovascular and cerebrovascular diseases
Other diseases
Overweight
Primary school
Drinker
URBMI, NRCMI, or URCMI
Middle school
Male
Smoker
UEBMI
High school
Other types of insurance
Variable
HR
B
Risk Factors for Outpatient CAP Cases
Effect Harmful Protective
Respiratory diseases
Three and more comorbidities
51−60 years
> 70 years
Obese
61−70 years
Two comorbidities
Underweight
One comorbidity
Cancer
Diabetes
Cardiovascular and cerebrovascular diseases
Overweight
Other diseases
Middle school
Primary school
Drinker
URBMI, NRCMI, or URCMI
UEBMI
Male
Smoker
High school
Other types of insurance
Variable
HR
C
Risk Factors for Inpatient CAP Cases
Effect Harmful Protective
> 70 years
61−70 years
Three and more comorbidities
Respiratory diseases
Two comorbidities
51−60 years
Cancer
Cardiovascular and cerebrovascular diseases
One comorbidity
Underweight
Obese
Diabetes
Male
URBMI, NRCMI, or URCMI
Overweight
Drinker
Other diseases
Smoker
Other types of insurance
Primary school
UEBMI
Middle school
High school
Variable
HR
