# Supplementary material for: Incidence of community-acquired pneumonia among adults between 2016 and 2023: an observational cohort study
Source: Epidemiol Infect. 2026 Jan 6;154:e15. doi: 10.1017/S0950268825100897 (PMC12835933; doi:10.1017/S0950268825100897)
Supplement: Wang et al. supplementary material [file S0950268825100897sup001.zip › FS3.pdf]

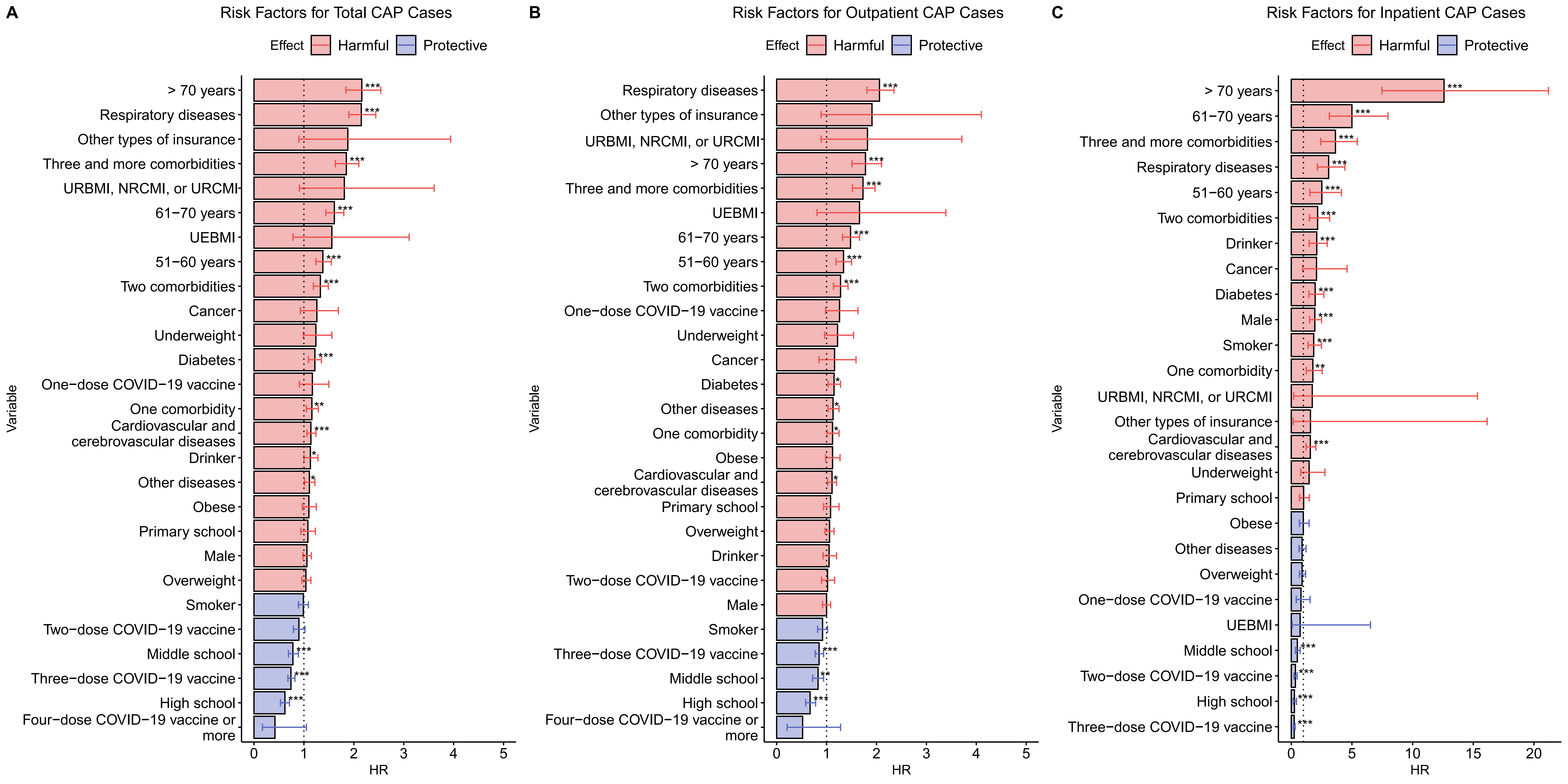
A
Risk Factors for Total CAP Cases
Effect Harmful Protective
> 70 years
Respiratory diseases
Other types of insurance
Three and more comorbidities
URBMI, NRCMI, or URCMI
61−70 years
UEBMI
51−60 years
Two comorbidities
Cancer
Underweight
Diabetes
One−dose COVID−19 vaccine
One comorbidity
Cardiovascular and cerebrovascular diseases
Drinker
Other diseases
Obese
Primary school
Male
Overweight
Smoker
Two−dose COVID−19 vaccine
Middle school
Three−dose COVID−19 vaccine
High school
Four−dose COVID−19 vaccine or more
Variable
HR
B
Risk Factors for Outpatient CAP Cases
Effect Harmful Protective
Respiratory diseases
Other types of insurance
URBMI, NRCMI, or URCMI
> 70 years
Three and more comorbidities
UEBMI
61−70 years
51−60 years
Two comorbidities
One−dose COVID−19 vaccine
Underweight
Cancer
Diabetes
Other diseases
One comorbidity
Obese
Cardiovascular and cerebrovascular diseases
Primary school
Overweight
Drinker
Two−dose COVID−19 vaccine
Male
Smoker
Three−dose COVID−19 vaccine
Middle school
High school
Four−dose COVID−19 vaccine or more
Variable
HR
C
Risk Factors for Inpatient CAP Cases
Effect Harmful Protective
> 70 years
61−70 years
Three and more comorbidities
Respiratory diseases
51−60 years
Two comorbidities
Drinker
Cancer
Diabetes
Male
Smoker
One comorbidity
URBMI, NRCMI, or URCMI
Other types of insurance
Cardiovascular and cerebrovascular diseases
Underweight
Primary school
Obese
Other diseases
Overweight
One−dose COVID−19 vaccine
UEBMI
Middle school
Two−dose COVID−19 vaccine
High school
Three−dose COVID−19 vaccine
Variable
HR
