# Supplementary material for: Incidence of community-acquired pneumonia among adults between 2016 and 2023: an observational cohort study
Source: Epidemiol Infect. 2026 Jan 6;154:e15. doi: 10.1017/S0950268825100897 (PMC12835933; doi:10.1017/S0950268825100897)
Supplement: Wang et al. supplementary material [file S0950268825100897sup001.zip › FS4.pdf]

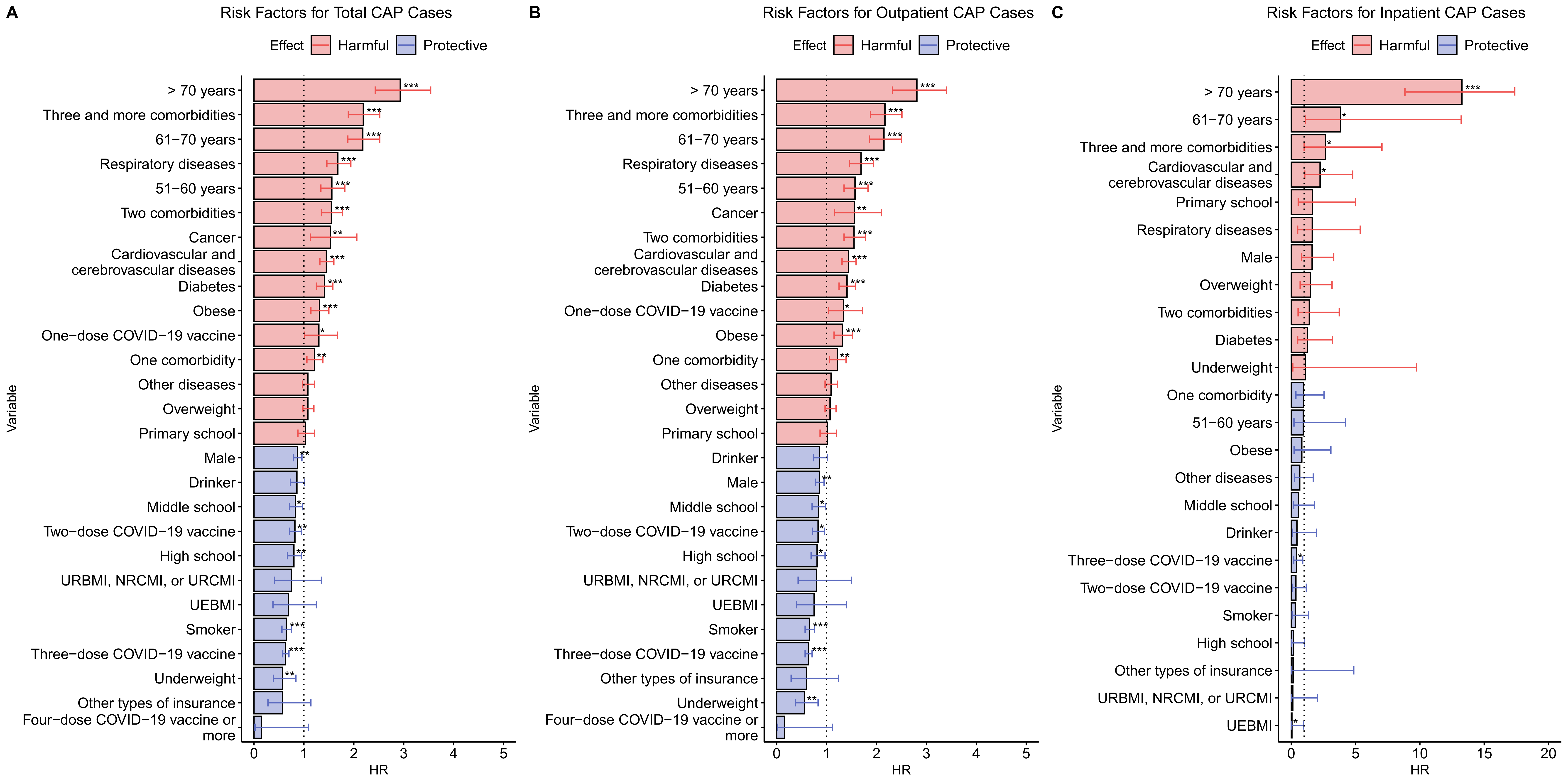
A
Risk Factors for Total CAP Cases
Effect Harmful Protective
> 70 years
Three and more comorbidities
61−70 years
Respiratory diseases
51−60 years
Two comorbidities
Cancer
Cardiovascular and cerebrovascular diseases
Diabetes
Obese
One−dose COVID−19 vaccine
One comorbidity
Other diseases
Overweight
Primary school
Male
Drinker
Middle school
Two−dose COVID−19 vaccine
High school
URBMI, NRCMI, or URCMI
UEBMI
Smoker
Three−dose COVID−19 vaccine
Underweight
Other types of insurance
Four−dose COVID−19 vaccine or more
Variable
HR
B
Risk Factors for Outpatient CAP Cases
Effect Harmful Protective
> 70 years
Three and more comorbidities
61−70 years
Respiratory diseases
51−60 years
Cancer
Two comorbidities
Cardiovascular and cerebrovascular diseases
Diabetes
One−dose COVID−19 vaccine
Obese
One comorbidity
Other diseases
Overweight
Primary school
Drinker
Male
Middle school
Two−dose COVID−19 vaccine
High school
URBMI, NRCMI, or URCMI
UEBMI
Smoker
Three−dose COVID−19 vaccine
Other types of insurance
Underweight
Four−dose COVID−19 vaccine or more
Variable
HR
C
Risk Factors for Inpatient CAP Cases
Effect Harmful Protective
> 70 years
61−70 years
Three and more comorbidities
Cardiovascular and cerebrovascular diseases
Primary school
Respiratory diseases
Male
Overweight
Two comorbidities
Diabetes
Underweight
One comorbidity
51−60 years
Obese
Other diseases
Middle school
Drinker
Three−dose COVID−19 vaccine
Two−dose COVID−19 vaccine
Smoker
High school
Other types of insurance
URBMI, NRCMI, or URCMI
UEBMI
Variable
HR
