# Supplementary material for: Incidence of community-acquired pneumonia among adults between 2016 and 2023: an observational cohort study
Source: Epidemiol Infect. 2026 Jan 6;154:e15. doi: 10.1017/S0950268825100897 (PMC12835933; doi:10.1017/S0950268825100897)
Supplement: Wang et al. supplementary material [file S0950268825100897sup001.zip › FS5.pdf]

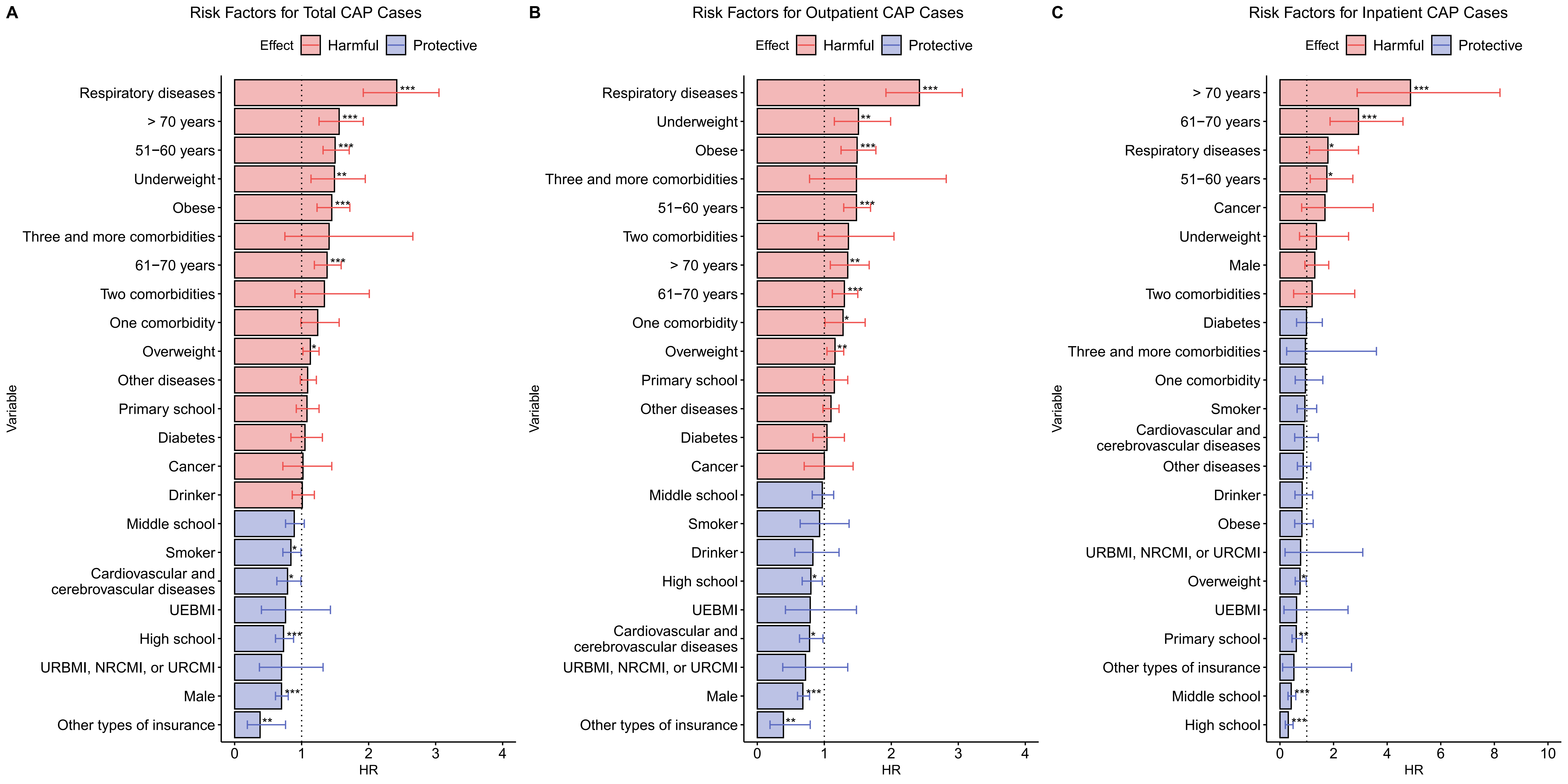
A
Risk Factors for Total CAP Cases
Effect Harmful Protective
Respiratory diseases ***
> 70 years ***
51−60 years ***
Underweight **
Obese ***
Three and more comorbidities
61−70 years ***
Two comorbidities
One comorbidity
Overweight *
Other diseases
Primary school
Diabetes
Cancer
Drinker
Middle school
Smoker *
Cardiovascular and cerebrovascular diseases *
UEBMI
High school ***
URBMI, NRCMI, or URCMI
Male ***
Other types of insurance **
Variable
HR
0 1 2 3 4
B
Risk Factors for Outpatient CAP Cases
Effect Harmful Protective
Respiratory diseases ***
Underweight **
Obese ***
Three and more comorbidities
51−60 years ***
Two comorbidities
> 70 years **
61−70 years ***
One comorbidity *
Overweight **
Primary school
Other diseases
Diabetes
Cancer
Middle school
Smoker
Drinker
High school *
UEBMI
Cardiovascular and cerebrovascular diseases *
URBMI, NRCMI, or URCMI
Male ***
Other types of insurance **
Variable
HR
0 1 2 3 4
C
Risk Factors for Inpatient CAP Cases
Effect Harmful Protective
> 70 years ***
61−70 years ***
Respiratory diseases *
51−60 years *
Cancer
Underweight
Male
Two comorbidities
Diabetes
Three and more comorbidities
One comorbidity
Smoker
Cardiovascular and cerebrovascular diseases
Other diseases
Drinker
Obese
URBMI, NRCMI, or URCMI
Overweight *
UEBMI
Primary school **
Other types of insurance
Middle school ***
High school ***
Variable
HR
0 2 4 6 8 10
