# Supplementary material for: Incidence of community-acquired pneumonia among adults between 2016 and 2023: an observational cohort study
Source: Epidemiol Infect. 2026 Jan 6;154:e15. doi: 10.1017/S0950268825100897 (PMC12835933; doi:10.1017/S0950268825100897)
Supplement: Wang et al. supplementary material [file S0950268825100897sup001.zip › FS6.pdf]

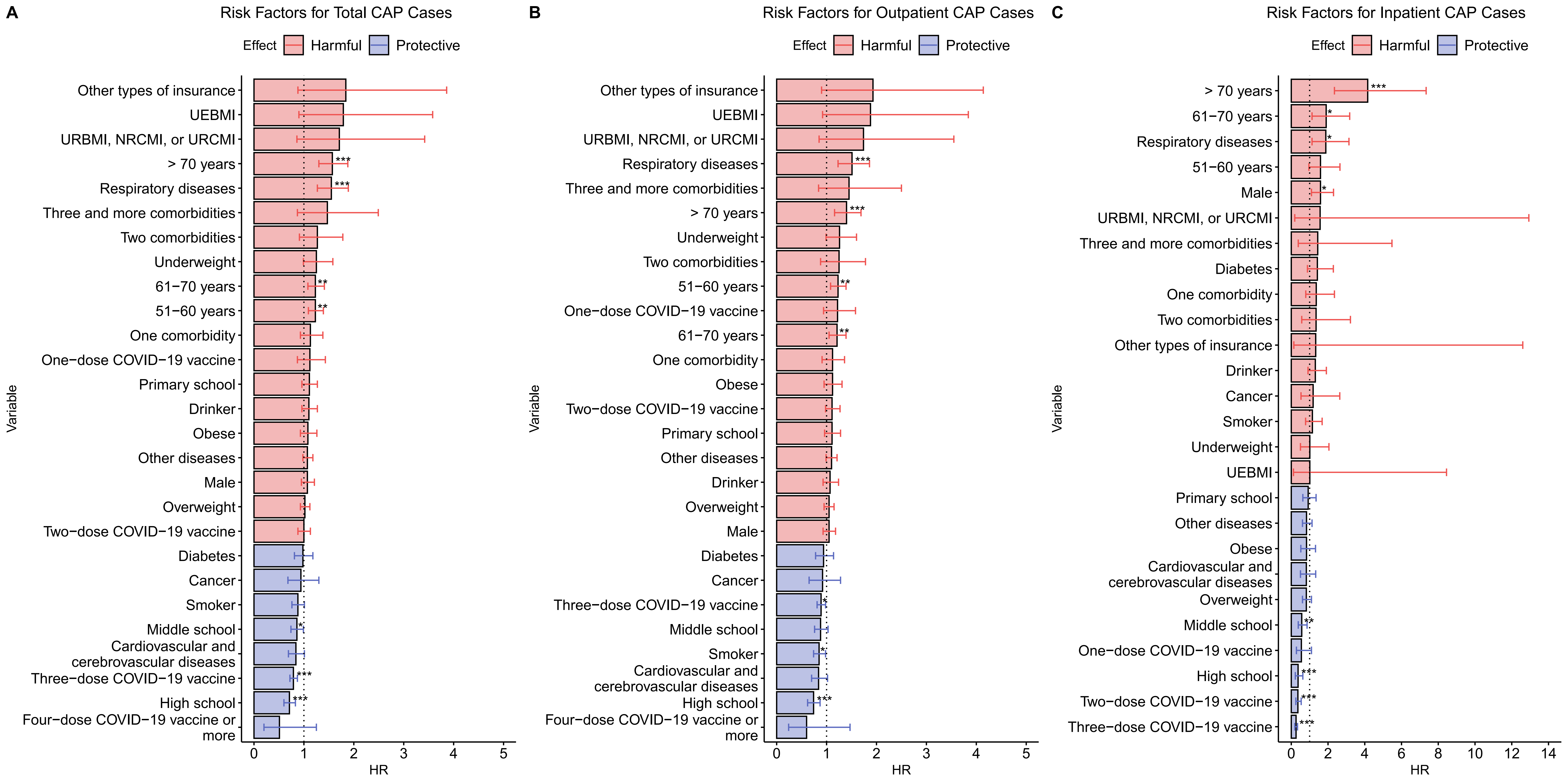

A
Risk Factors for Total CAP Cases
Effect
Harmful
Protective
Other types of insurance
UEBMI
URBMI, NRCMI, or URCMI
> 70 years
Respiratory diseases
Three and more comorbidities
Two comorbidities
Underweight
61−70 years
51−60 years
One comorbidity
One−dose COVID−19 vaccine
Primary school
Drinker
Obese
Other diseases
Male
Overweight
Two−dose COVID−19 vaccine
Diabetes
Cancer
Smoker
Middle school
Cardiovascular and cerebrovascular diseases
Three−dose COVID−19 vaccine
High school
Four−dose COVID−19 vaccine or more
Variable
HR
B
Risk Factors for Outpatient CAP Cases
Effect
Harmful
Protective
Other types of insurance
UEBMI
URBMI, NRCMI, or URCMI
Respiratory diseases
Three and more comorbidities
> 70 years
Underweight
Two comorbidities
51−60 years
One−dose COVID−19 vaccine
61−70 years
One comorbidity
Obese
Two−dose COVID−19 vaccine
Primary school
Other diseases
Drinker
Overweight
Male
Diabetes
Cancer
Three−dose COVID−19 vaccine
Middle school
Smoker
Cardiovascular and cerebrovascular diseases
High school
Four−dose COVID−19 vaccine or more
Variable
HR
C
Risk Factors for Inpatient CAP Cases
Effect
Harmful
Protective
> 70 years
61−70 years
Respiratory diseases
51−60 years
Male
URBMI, NRCMI, or URCMI
Three and more comorbidities
Diabetes
One comorbidity
Two comorbidities
Other types of insurance
Drinker
Cancer
Smoker
Underweight
UEBMI
Primary school
Other diseases
Obese
Cardiovascular and cerebrovascular diseases
Overweight
Middle school
One−dose COVID−19 vaccine
High school
Two−dose COVID−19 vaccine
Three−dose COVID−19 vaccine
Variable
HR
