# Supplementary material for: Incidence of community-acquired pneumonia among adults between 2016 and 2023: an observational cohort study
Source: Epidemiol Infect. 2026 Jan 6;154:e15. doi: 10.1017/S0950268825100897 (PMC12835933; doi:10.1017/S0950268825100897)
Supplement: Wang et al. supplementary material [file S0950268825100897sup001.zip › FS7.pdf]

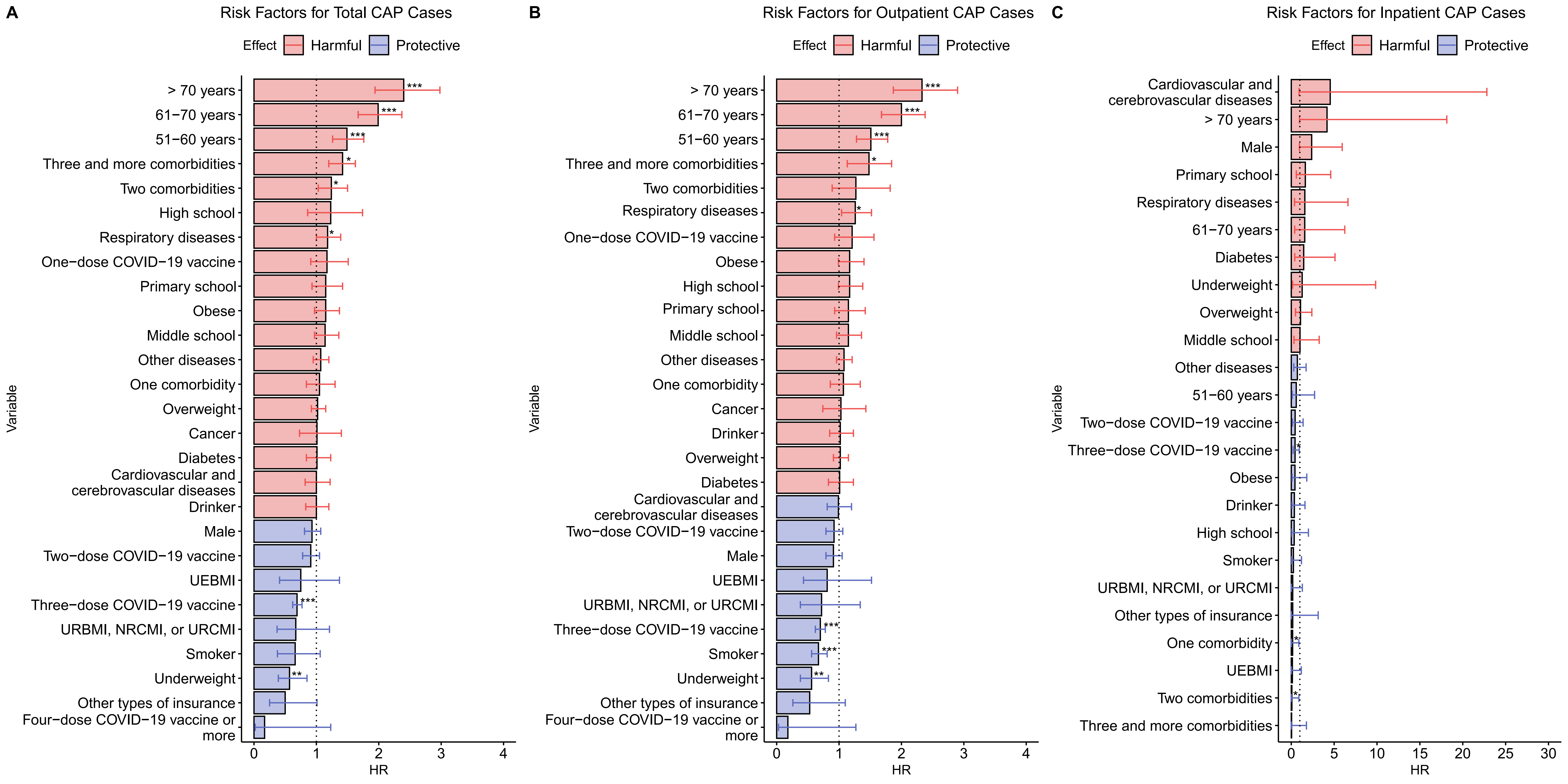

A
Risk Factors for Total CAP Cases
Effect Harmful Protective
> 70 years
61−70 years
51−60 years
Three and more comorbidities
Two comorbidities
High school
Respiratory diseases
One−dose COVID−19 vaccine
Primary school
Obese
Middle school
Other diseases
One comorbidity
Overweight
Cancer
Diabetes
Cardiovascular and cerebrovascular diseases
Drinker
Male
Two−dose COVID−19 vaccine
UEBMI
Three−dose COVID−19 vaccine
URBMI, NRCMI, or URCMI
Smoker
Underweight
Other types of insurance
Four−dose COVID−19 vaccine or more
Variable
HR
B
Risk Factors for Outpatient CAP Cases
Effect Harmful Protective
> 70 years
61−70 years
51−60 years
Three and more comorbidities
Two comorbidities
Respiratory diseases
One−dose COVID−19 vaccine
Obese
High school
Primary school
Middle school
Other diseases
One comorbidity
Cancer
Drinker
Overweight
Diabetes
Cardiovascular and cerebrovascular diseases
Two−dose COVID−19 vaccine
Male
UEBMI
URBMI, NRCMI, or URCMI
Three−dose COVID−19 vaccine
Smoker
Underweight
Other types of insurance
Four−dose COVID−19 vaccine or more
Variable
HR
C
Risk Factors for Inpatient CAP Cases
Effect Harmful Protective
Cardiovascular and cerebrovascular diseases
> 70 years
Male
Primary school
Respiratory diseases
61−70 years
Diabetes
Underweight
Overweight
Middle school
Other diseases
51−60 years
Two−dose COVID−19 vaccine
Three−dose COVID−19 vaccine
Obese
Drinker
High school
Smoker
URBMI, NRCMI, or URCMI
Other types of insurance
One comorbidity
UEBMI
Two comorbidities
Three and more comorbidities
Variable
HR
