# Supplementary material for: Incidence of community-acquired pneumonia among adults between 2016 and 2023: an observational cohort study
Source: Epidemiol Infect. 2026 Jan 6;154:e15. doi: 10.1017/S0950268825100897 (PMC12835933; doi:10.1017/S0950268825100897)
Supplement: Wang et al. supplementary material [file S0950268825100897sup001.zip › Supplementary materials-clean.docx]

**Supplementary materials**

**Table S1. Overall incidence of first-time CAP by baseline characteristics**

**Table S2. Inpatient CAP incidence across various NPI stages among subpopulations.**

**Table S3. Outpatient CAP incidence across various NPI stages among subpopulations.**

**Fig. S1 Annual incidence rate of CAP standardized with age and sex stratified by district**

**Fig. S2 Risk factors of CAP before NPIs using univariate models.**

**Fig. S3 Risk factors of CAP during NPIs using univariate models.**

**Fig. S4 Risk factors of CAP after NPIs using univariate models.**

**Fig. S5 Risk factors of CAP before NPIs using multivariate models.**

**Fig. S6 Risk factors of CAP during NPIs using multivariate models.**

**Fig. S7 Risk factors of CAP after NPIs using multivariate models.**

**Table S1. Overall incidence of first-time CAP by baseline characteristics**

| Characteristics | Incidence density of CAP episodes (/1000 person-years) | Hospitalization density of CAP episodes (/1000 person-years) | Outpatient density of CAP episodes (/1000 person-years) |
| --- | --- | --- | --- |
| **Sex** |  |  |  |
| Male | 25.0 (24.1-25.9) | 2.4 (2.1-2.7) | 22.6 (21.8-23.5) |
| Female | 28.5 (27.7-29.3) | 1.8 (1.6-2.0) | 26.7 (25.9-27.4) |
| **Age at baseline** |  |  |  |
| 20-50 years | 18.8 (17.8-19.8) | 0.6 (0.4-0.8) | 18.2 (17.2-19.2) |
| 51-60 years | 27.4 (26.3-28.4) | 1.4 (1.2-1.7) | 25.9 (25.0-27.0) |
| 61-70 years | 30.6 (29.6-31.7) | 2.9 (2.6-3.3) | 27.7 (26.7-28.7) |
| > 70 years | 36.4 (33.9-39.1) | 5.5 (4.6-6.6) | 30.9 (28.5-33.3) |
| **Education level** |  |  |  |
| No formal school | 32.3 (30.3-34.3) | 3.9 (3.3-4.7) | 28.3 (26.5-30.2) |
| Primary school | 33.0 (31.8-34.3) | 3.1 (2.7-3.5) | 30.0 (28.8-31.2) |
| Middle school | 25.6 (24.7-26.5) | 1.6 (1.3-1.8) | 24.1 (23.2-25.0) |
| High school | 21.0 (20.0-22.1) | 1.0 (0.8-1.2) | 20.1 (19.1-21.1) |
| **Medical insurance** |  |  |  |
| Uninsured | 31.4 (23.1-41.5) | 3.5 (1.3-7.5) | 27.9 (20.1-37.5) |
| UEBMI | 24.4 (23.5-25.4) | 1.2 (1.0-1.4) | 23.2 (22.3-24.2) |
| URBMIRCMI, or URCMI | 28.5 (27.8-29.3) | 2.5 (2.2-2.7) | 26.1 (25.4-26.8) |
| Other types of insurance | 21.2 (17.8-24.9) | 1.7 (0.9-3.0) | 19.4 (16.2-23.1) |
| **BMI group** |  |  |  |
| Underweight | 28.9 (25.4-32.6) | 1.5 (0.8-2.5) | 27.3 (24.0-31.0) |
| Normal | 25.6 (24.8-26.4) | 2.0 (1.7-2.2) | 23.6 (22.8-24.4) |
| Overweight | 27.5 (26.5-28.4) | 2.0 (1.8-2.3) | 25.4 (24.5-26.4) |
| Obese | 31.0 (29.3-32.9) | 2.5 (2.0-3.0) | 28.5 (26.8-30.3) |
| **Smoking** |  |  |  |
| Non-smoker | 27.9 (27.3-28.6) | 2.0 (1.8-2.2) | 26.0 (25.3-26.6) |
| Smoker | 23.3 (22.1-24.6) | 2.3 (2.0-2.8) | 21.0 (19.8-22.2) |
| **Drinking** |  |  |  |
| Non-drinker | 27.2 (26.6-27.8) | 2.0 (1.8-2.2) | 25.2 (24.6-25.8) |
| Drinker | 26.1 (24.4-27.9) | 2.6 (2.1-3.2) | 23.5 (21.9-25.2) |
| **Underlying conditions** |  |  |  |
| Cardiovascular and cerebrovascular diseases | 28.9 (28.1-29.8) | 2.5 (2.3-2.8) | 26.4 (25.6-27.2) |
| Respiratory diseases | 50.7 (48.0-53.6) | 4.2 (3.5-5.1) | 46.5 (43.9-49.2) |
| Diabetes | 31.7 (30.1-33.3) | 3.1 (2.6-3.6) | 28.6 (27.1-30.1) |
| Cancer | 34.8 (29.9-40.0) | 3.1 (1.8-4.8) | 31.7 (27.1-36.8) |
| Other diseases | 28.0 (27.1-29.0) | 2.2 (1.9-2.5) | 25.8 (24.9-26.8) |
| **Comorbidity** |  |  |  |
| None | 22.4 (21.4-23.4) | 1.3 (1.1-1.6) | 21.0 (20.1-22.0) |
| One | 25.8 (24.8-26.8) | 1.7 (1.5-2.0) | 24.1 (23.1-25.0) |
| Two | 29.2 (28.0-30.5) | 2.5 (2.2-2.9) | 26.7 (25.5-27.9) |
| Three and more | 37.3 (35.4-39.2) | 3.6 (3.0-4.2) | 33.7 (31.9-35.6) |

**Table S2. Inpatient CAP incidence across various NPI stages among subpopulations.**

| Characteristic | CAP incidence before NPIs (/1000 person-years) | CAP incidence during NPIs (/1000 person-years) | CAP incidence after NPIs (/1000 person-years) | IRR for During vs Before | P value for During vs Before | IRR for After vs During | P value for After vs During |
| --- | --- | --- | --- | --- | --- | --- | --- |
| **Sex** |  |  |  |  |  |  |  |
| Male | 3.5 (3.0-4.0) | 3.0 (2.6-3.4) | 2.6 (1.6-4.0) | 0.87 (0.55-1.36) | 0.54 | 0.86 (0.54-1.35) | 0.51 |
| Female | 3.2 (2.9-3.6) | 1.6 (1.3-1.8) | 1.7 (1.0-2.6) | 0.49 (0.30-0.80) | < 0.01 | 1.05 (0.64-1.74) | 0.84 |
| **Age at baseline** |  |  |  |  |  |  |  |
| 20-50 years | 1.0 (0.7-1.3) | 0.6 (0.4-0.9) | 0.7 (0.2-1.9) | 0.50 (0.12-2.10) | 0.35 | 1.53 (0.36-6.42) | 0.56 |
| 51-60 years | 2.2 (1.8-2.7) | 1.4 (1.1-1.8) | 0.7 (0.2-1.6) | 0.62 (0.30-1.27) | 0.19 | 0.49 (0.23-1.06) | 0.07 |
| 61-70 years | 5.1 (4.5-5.8) | 2.8 (2.4-3.3) | 2.8 (1.7-4.3) | 0.55 (0.34-0.89) | < 0.05 | 1.00 (0.61-1.63) | 1 |
| > 70 years | 9.2 (7.3-11.4) | 7.0 (5.6-8.6) | 9.0 (4.7-15.4) | 0.80 (0.40-1.59) | 0.53 | 1.22 (0.62-2.42) | 0.56 |
| **Education level** |  |  |  |  |  |  |  |
| No formal school | 7.7 (6.4-9.2) | 3.4 (2.6-4.3) | 2.7 (1.0-5.7) | 0.42 (0.18-0.94) | < 0.05 | 0.84 (0.36-1.96) | 0.69 |
| Primary school | 4.5 (3.9-5.2) | 3.4 (2.8-3.9) | 3.8 (2.3-5.8) | 0.74 (0.45-1.24) | 0.26 | 1.13 (0.68-1.88) | 0.65 |
| Middle school | 2.3 (2.0-2.8) | 1.7 (1.4-2.0) | 1.6 (0.8-2.7) | 0.75 (0.41-1.35) | 0.33 | 0.92 (0.50-1.67) | 0.78 |
| High school | 1.3 (1.0-1.8) | 0.9 (0.6-1.2) | 0.5 (0.1-1.5) | 0.73 (0.26-2.04) | 0.55 | 0.51 (0.17-1.51) | 0.23 |
| **Medical insurance** |  |  |  |  |  |  |  |
| Uninsured | 4.0 (1.0-10.4) | 1.3 (0.1-5.8) | 12.6 (0.7-55.4) | 1.35 (0.07-26.08) | 1 | 2.20 (0.13-37.55) | 1 |
| UEBMI | 1.9 (1.5-2.3) | 1.1 (0.8-1.4) | 1.1 (0.4-2.1) | 0.57 (0.25-1.30) | 0.18 | 1.00 (0.43-2.32) | 1 |
| URBMI-NRCMI-or URCMI | 4.1 (3.7-4.5) | 2.6 (2.3-3.0) | 2.4 (1.6-3.5) | 0.64 (0.44-0.94) | < 0.05 | 0.93 (0.64-1.36) | 0.72 |
| Other types of insurance | 2.5 (1.1-4.8) | 2.4 (1.1-4.5) | 2.8 (0.2-12.2) | 1.11 (0.14-9.04) | 0.92 | 0.98 (0.12-7.77) | 0.99 |
| **BMI group** |  |  |  |  |  |  |  |
| Underweight | 4.7 (2.8-7.1) | 4.2 (2.6-6.4) | 2.0 (0.1-8.8) | 0.86 (0.20-3.73) | 0.84 | 0.50 (0.11-2.29) | 0.37 |
| Normal | 3.2 (2.8-3.7) | 2.2 (1.8-2.5) | 1.7 (1.0-2.8) | 0.65 (0.40-1.07) | 0.09 | 0.83 (0.50-1.37) | 0.46 |
| Overweight | 3.1 (2.7-3.6) | 1.9 (1.6-2.3) | 2.6 (1.6-4.0) | 0.61 (0.35-1.07) | 0.09 | 1.38 (0.78-2.43) | 0.26 |
| Obese | 4.2 (3.3-5.2) | 2.2 (1.6-2.9) | 1.4 (0.3-3.5) | 0.54 (0.22-1.35) | 0.19 | 0.61 (0.24-1.58) | 0.31 |
| **Smoking** |  |  |  |  |  |  |  |
| Non-smoker | 3.5 (3.1-3.8) | 1.9 (1.7-2.1) | 2.6 (1.9-3.5) | 0.55 (0.47-0.64) | <0.001 | 1.39 (1.00-1.92) | <0.05 |
| Smoker | 2.9 (2.3-3.6) | 3.0 (2.5-3.7) | 0.9 (0.2-2.3) | 1.06 (0.78-1.43) | 0.72 | 0.29 (0.09-0.92) | <0.05 |
| **Drinking** |  |  |  |  |  |  |  |
| Non-drinker | 3.4 (3.1-3.7) | 1.9 (1.7-2.2) | 2.4 (1.8-3.2) | 0.57 (0.49-0.66) | <0.001 | 1.26 (0.91-1.74) | 0.16 |
| Drinker | 3.1 (2.3-4.1) | 3.6 (2.8-4.5) | 1.5 (0.4-3.8) | 1.14 (0.79-1.65) | 0.48 | 0.42 (0.13-1.32) | 0.14 |
| **Underlying conditions** |  |  |  |  |  |  |  |
| Cardiovascular and cerebrovascular diseases | 4.0 (3.6-4.5) | 2.6 (2.3-2.9) | 3.1 (2.2-4.3) | 0.64 (0.54-0.76) | <0.001 | 1.21 (0.83-1.74) | 0.32 |
| Respiratory diseases | 8.8 (7.2-10.7) | 4.7 (3.8-5.8) | 3.7 (1.6-7.1) | 0.54 (0.40-0.72) | <0.001 | 0.77 (0.36-1.67) | 0.51 |
| Diabetes | 4.0 (3.2-5.0) | 3.5 (2.9-4.2) | 2.6 (1.2-4.8) | 0.86 (0.68-1.10) | 0.34 | 0.75 (0.55-1.00) | 0.41 |
| Cancer | 7.6 (4.4-11.9) | 4.8 (2.8-7.5) | 2.8 (0.2-12.2) | 0.62 (0.34-1.11) | 0.2 | 0.07 (0.01-0.53) | 0.6 |
| Other diseases | 3.8 (2.2-4.2) | 2.0 (1.7-2.3) | 3.1 (2.0-4.5) | 0.52 (0.42-0.66) | <0.001 | 1.57 (1.01-2.44) | <0.05 |
| **Comorbidity** |  |  |  |  |  |  |  |
| None | 2.0 (1.6-2.5) | 1.1 (0.9-1.5) | 1.6 (0.7-2.9) | 0.58 (0.25-1.32) | 0.19 | 1.32 (0.58-3.04) | 0.51 |
| One | 2.8 (2.3-3.3) | 2.0 (1.7-2.4) | 1.5 (0.7-2.7) | 0.72 (0.40-1.30) | 0.27 | 0.74 (0.41-1.37) | 0.34 |
| Two | 4.4 (3.7-5.2) | 2.5 (2.0-3.0) | 2.1 (1.0-3.9) | 0.59 (0.31-1.11) | 0.1 | 0.82 (0.44-1.54) | 0.54 |
| Three and more | 6.2 (5.0-7.5) | 4.0 (3.2-4.9) | 4.4 (2.2-7.6) | 0.64 (0.33-1.24) | 0.18 | 1.11 (0.56-2.19) | 0.77 |

**Table S3. Outpatient CAP incidence across NPI stages among subpopulations.**

| Characteristics | CAP incidence before NPIs (/1000 person-years) | CAP incidence during NPIs (/1000 person-years) | CAP incidence after NPIs (/1000 person-years) | IRR for During vs Before | P value for During vs Before | IRR for After vs During | P value for After vs During |
| --- | --- | --- | --- | --- | --- | --- | --- |
| **Sex** |  |  |  |  |  |  |  |
| Male | 39.6 (38.0-41.3) | 23.0 (21.8-24.2) | 89.3 (82.4-96.6) | 0.58 (0.54-0.62) | < 0.001 | 3.89 (3.53-4.28) | < 0.001 |
| Female | 52.8 (51.2-54.4) | 23.7 (22.7-24.7) | 103.7 (97.7-109.9) | 0.45 (0.43-0.47) | < 0.001 | 4.38 (4.06-4.71) | < 0.001 |
| **Age at baseline** | |  |  |  |  |  |  |
| 20-50 years | 35.3 (33.4-37.4) | 17.4 (16.1-18.7) | 56.3 (49.3-63.8) | 0.49 (0.45-0.54) | < 0.001 | 3.24 (2.78-3.77) | < 0.001 |
| 51-60 years | 52.0 (50.0-54.1) | 23.2 (21.9-24.5) | 88.6 (81.2-96.4) | 0.44 (0.41-0.48) | < 0.001 | 3.83 (3.44-4.25) | < 0.001 |
| 61-70 years | 50.4 (48.4-52.4) | 25.7 (24.4-27.0) | 121.9 (113.5-130.6) | 0.51 (0.48-0.54) | < 0.001 | 4.75 (4.34-5.19) | < 0.001 |
| > 70 years | 54.1 (49.4-59.2) | 33.0 (29.7-36.4) | 159.8 (138.4-183.2) | 0.61 (0.53-0.70) | < 0.001 | 4.85 (4.04-5.82) | < 0.001 |
| **Education level** |  |  |  |  |  |  |  |
| No formal school | 52.3 (48.7-56.0) | 28.0 (25.6-30.6) | 109.4 (95.1-125.0) | 0.54 (0.48-0.60) | < 0.001 | 3.90 (3.30-4.61) | < 0.001 |
| Primary school | 54.5 (52.2-56.8) | 29.4 (27.8-31.0) | 111.4 (102.2-121.1) | 0.54 (0.50-0.58) | < 0.001 | 3.79 (3.42-4.20) | < 0.001 |
| Middle school | 46.6 (44.8-48.5) | 21.8 (20.7-23.0) | 91.2 (84.2-98.5) | 0.47 (0.44-0.50) | < 0.001 | 4.18 (3.79-4.61) | < 0.001 |
| High school | 37.3 (35.1-39.6) | 17.2 (15.9-18.5) | 88.9 (80.1-98.4) | 0.46 (0.41-0.51) | < 0.001 | 5.19 (4.53-5.93) | < 0.001 |
| **Medical insurance** |  |  |  |  |  |  |  |
| Uninsured | 49.7 (35.3-67.5) | 13.3 (6.6-23.3) | 126.0 (63.1-221.0) | 0.27 (0.14-0.55) | < 0.001 | 9.43 (3.92-22.66) | < 0.001 |
| UEBMI | 46.0 (44.0-48.1) | 21.7 (20.4-23.0) | 94.0 (86.2-102.2) | 0.47 (0.44-0.51) | < 0.001 | 4.34 (3.90-4.83) | < 0.001 |
| URBMI-NRCMI-or URCMI | 48.7 (47.2-50.1) | 24.4 (23.4-25.3) | 100.6 (94.9-106.5) | 0.50 (0.48-0.53) | < 0.001 | 4.13 (3.85-4.43) | < 0.001 |
| Other types of insurance | 29.6 (23.7-36.4) | 23.4 (18.5-29.0) | 75.0 (50.2-107.0) | 0.80 (0.58-1.09) | 0.16 | 3.19 (2.04-4.99) | < 0.001 |
| **BMI group** |  |  |  |  |  |  |  |
| Underweight | 54.7 (47.7-62.4) | 28.3 (23.7-33.5) | 52.2 (34.6-74.9) | 0.52 (0.41-0.65) | < 0.001 | 1.84 (1.20-2.82) | < 0.01 |
| Normal | 44.4 (42.8-46.1) | 22.4 (21.3-23.5) | 92.8 (86.3-99.6) | 0.50 (0.47-0.54) | < 0.001 | 4.15 (3.80-4.54) | < 0.001 |
| Overweight | 47.8 (46.0-49.7) | 23.4 (22.3-24.6) | 99.7 (92.4-107.3) | 0.49 (0.46-0.52) | < 0.001 | 4.25 (3.88-4.67) | < 0.001 |
| Obese | 56.0 (52.5-59.7) | 26.1 (24.0-28.4) | 122.6 (108.6-137.8) | 0.47 (0.42-0.52) | < 0.001 | 4.69 (4.03-5.46) | < 0.001 |
| **Smoking** |  |  |  |  |  |  |  |
| Non-smoker | 50.4 (49.1-51.8) | 22.5 (21.7-23.3) | 99.2 (94.4-104.2) | 0.45 (0.43-0.47) | < 0.001 | 4.41 (4.15-4.68) | < 0.001 |
| Smoker | 35.5 (33.3-37.8) | 20.2 (18.6-21.8) | 67.3 (59.0-76.4) | 0.57 (0.51-0.63) | < 0.001 | 3.33 (2.87-3.88) | < 0.001 |
| **Drinking** |  |  |  |  |  |  |  |
| Non-drinker | 48.4 (47.1-49.6) | 21.8 (21.1-22.6) | 94.7 (90.1-99.3) | 0.45 (0.43-0.47) | < 0.001 | 4.33 (4.09-4.60) | < 0.001 |
| Drinker | 40.5 (37.4-43.7) | 24.3 (22.1-26.6) | 84.7 (72.6-98.0) | 0.60 (0.53-0.68) | < 0.001 | 3.49 (2.93-4.16) | < 0.001 |
| **Underlying conditions** |  |  |  |  |  |  |  |
| Cardiovascular and cerebrovascular diseases | 50.5 (48.8-52.2) | 23.3 (22.3-24.3) | 109.5 (103.2-116.0) | 0.46 (0.44-0.49) | < 0.001 | 4.71 (4.38-5.06) | < 0.001 |
| Respiratory diseases | 109.6 (103.6-115.8) | 42.4 (39.4-45.5) | 148.6 (132.0-166.6) | 0.39 (0.35-0.42) | < 0.001 | 3.51 (3.06-4.02) | < 0.001 |
| Diabetes | 53.7 (50.5-56.9) | 23.6 (21.9-25.4) | 122.5 (110.5-135.2) | 0.46 (0.43-0.49) | < 0.001 | 5.13 (4.68-5.63) | < 0.001 |
| Cancer | 61.5 (51.5-72.7) | 22.2 (17.5-27.6) | 136.4 (101.7-178.2) | 0.38 (0.31-0.47) | < 0.001 | 6.16 (4.72-8.05) | < 0.001 |
| Other diseases | 49.4 (47.4-51.4) | 22.5 (21.4-23.6) | 97.1 (90.2-104.4) | 0.46 (0.43-0.48) | < 0.001 | 4.32 (3.96-4.72) | < 0.001 |
| **Comorbidity** |  |  |  |  |  |  |  |
| None | 38.1 (36.3-40.0) | 19.0 (17.7-20.2) | 72.0 (64.9-79.6) | 0.50 (0.46-0.54) | < 0.001 | 3.80 (3.36-4.30) | < 0.001 |
| One | 45.8 (43.9-47.8) | 22.0 (20.8-23.3) | 87.7 (80.4-95.4) | 0.48 (0.45-0.52) | < 0.001 | 3.99 (3.59-4.43) | < 0.001 |
| Two | 50.8 (48.3-53.3) | 25.1 (23.5-26.7) | 111.9 (102.1-122.3) | 0.49 (0.44-0.55) | < 0.001 | 4.46 (3.91-5.10) | < 0.001 |
| Three and more | 69.7 (65.7-73.8) | 34.1 (31.7-36.7) | 157.6 (142.0-174.5) | 0.49 (0.45-0.53) | < 0.001 | 4.62 (4.11-5.21) | < 0.001 |


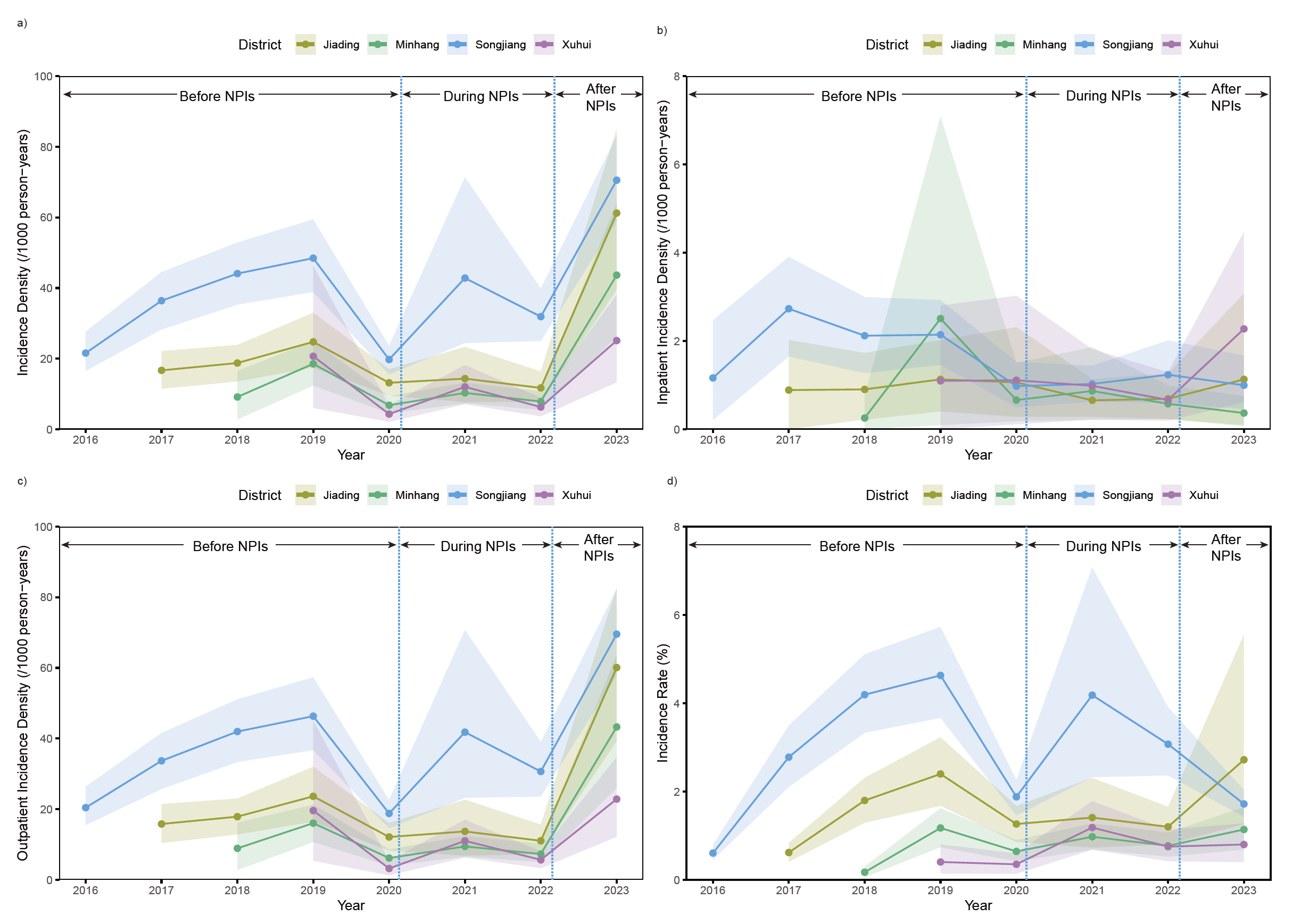


**Fig. S1 Annual incidence rate of CAP standardized with age and sex stratified by district**


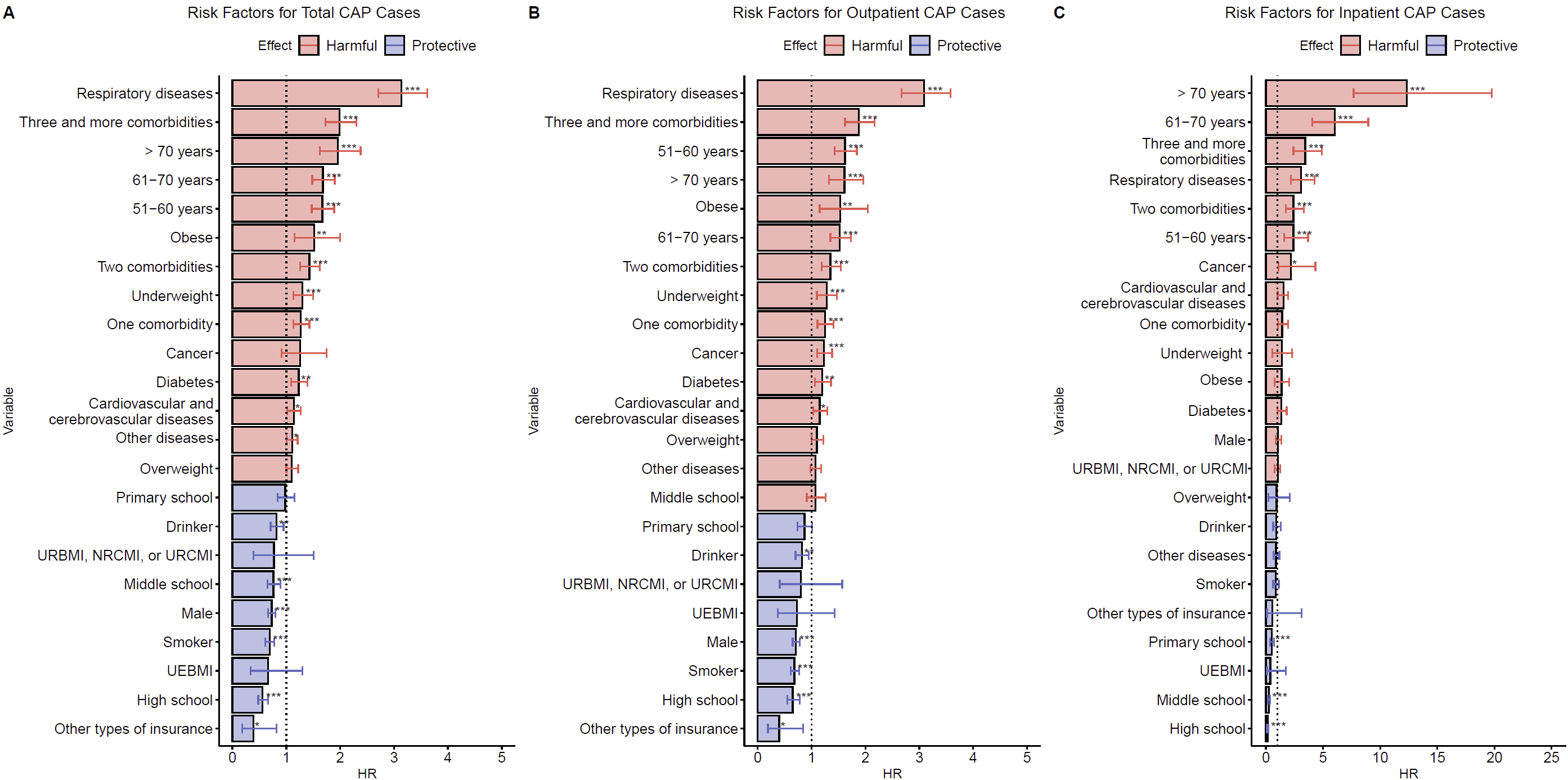
 **Fig. S2 Risk factors of CAP before NPIs using univariate models.**


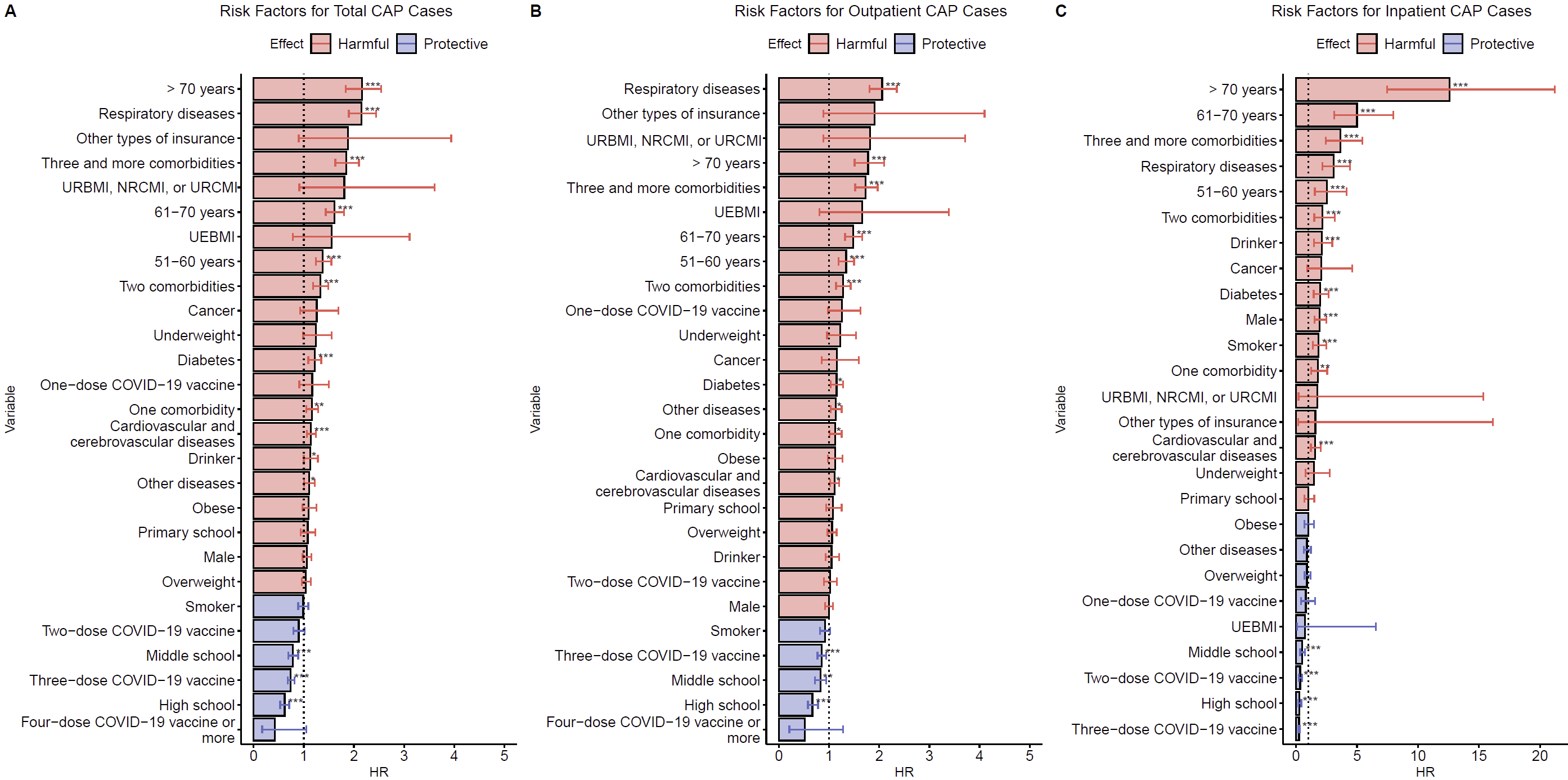
 **Fig. S3 Risk factors of CAP during NPIs using univariate models.**


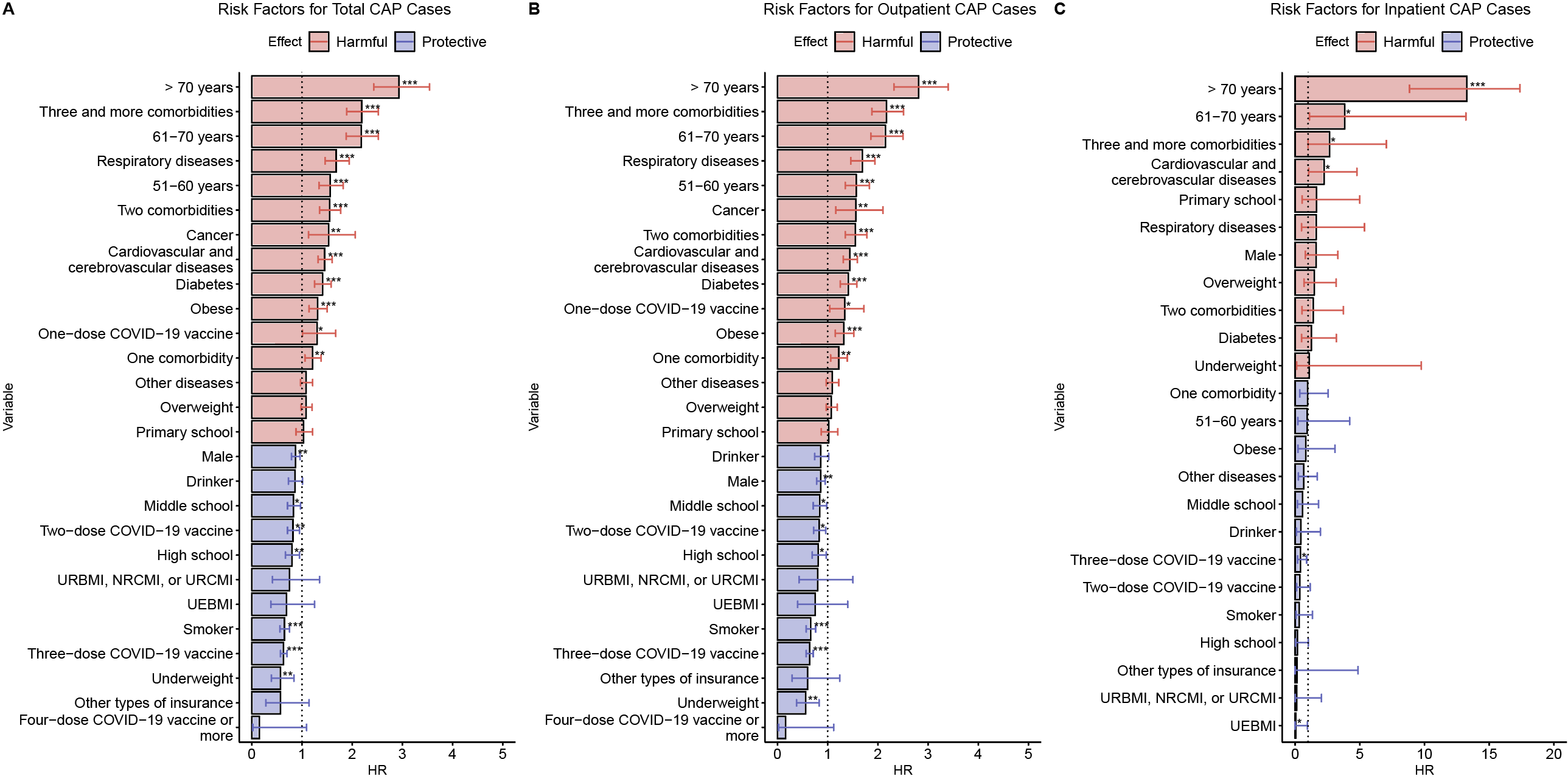
 **Fig. S4 Risk factors of CAP after NPIs using univariate models.**


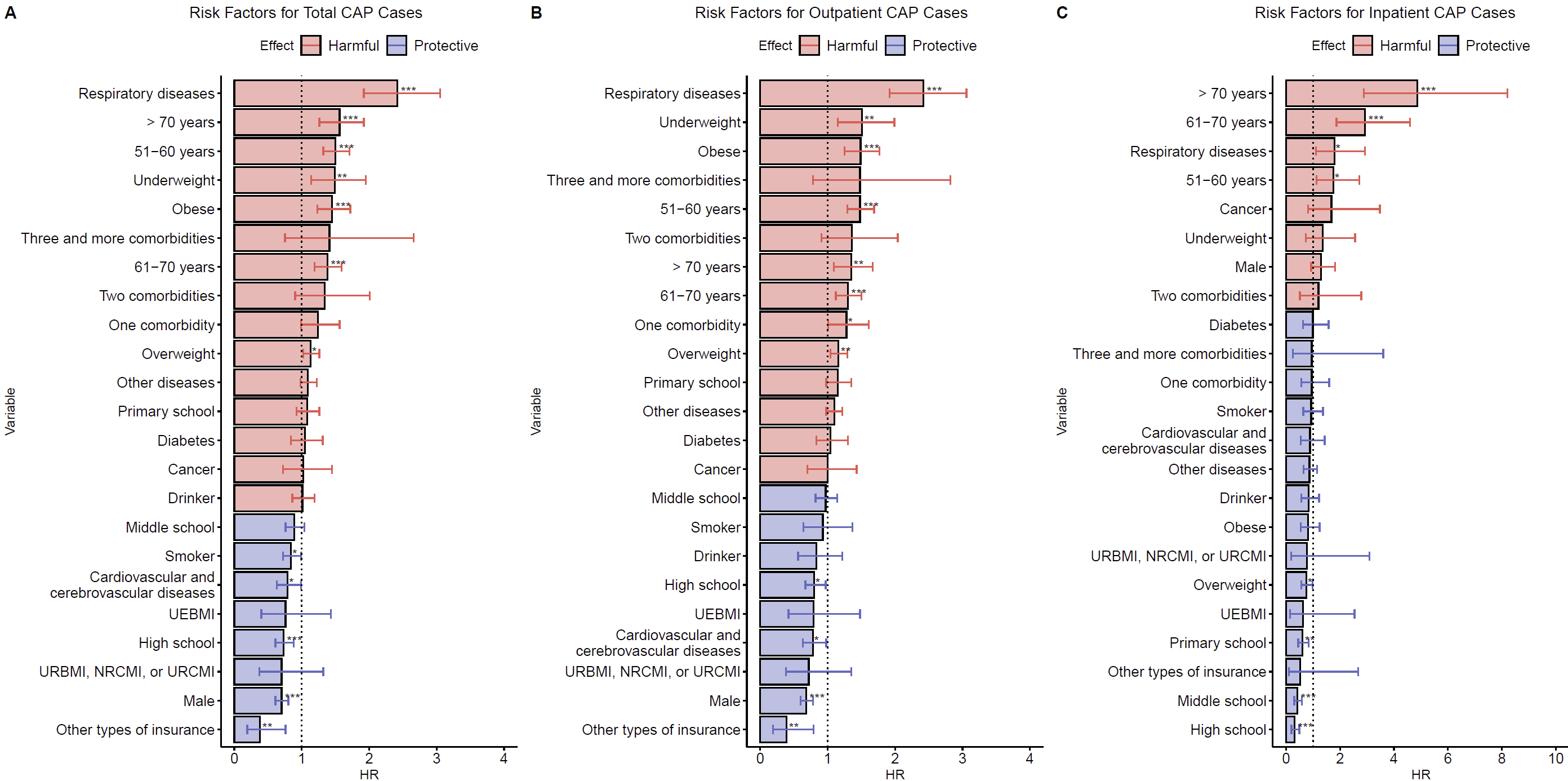
 **Fig. S5 Risk factors of CAP before NPIs using multivariate models.**


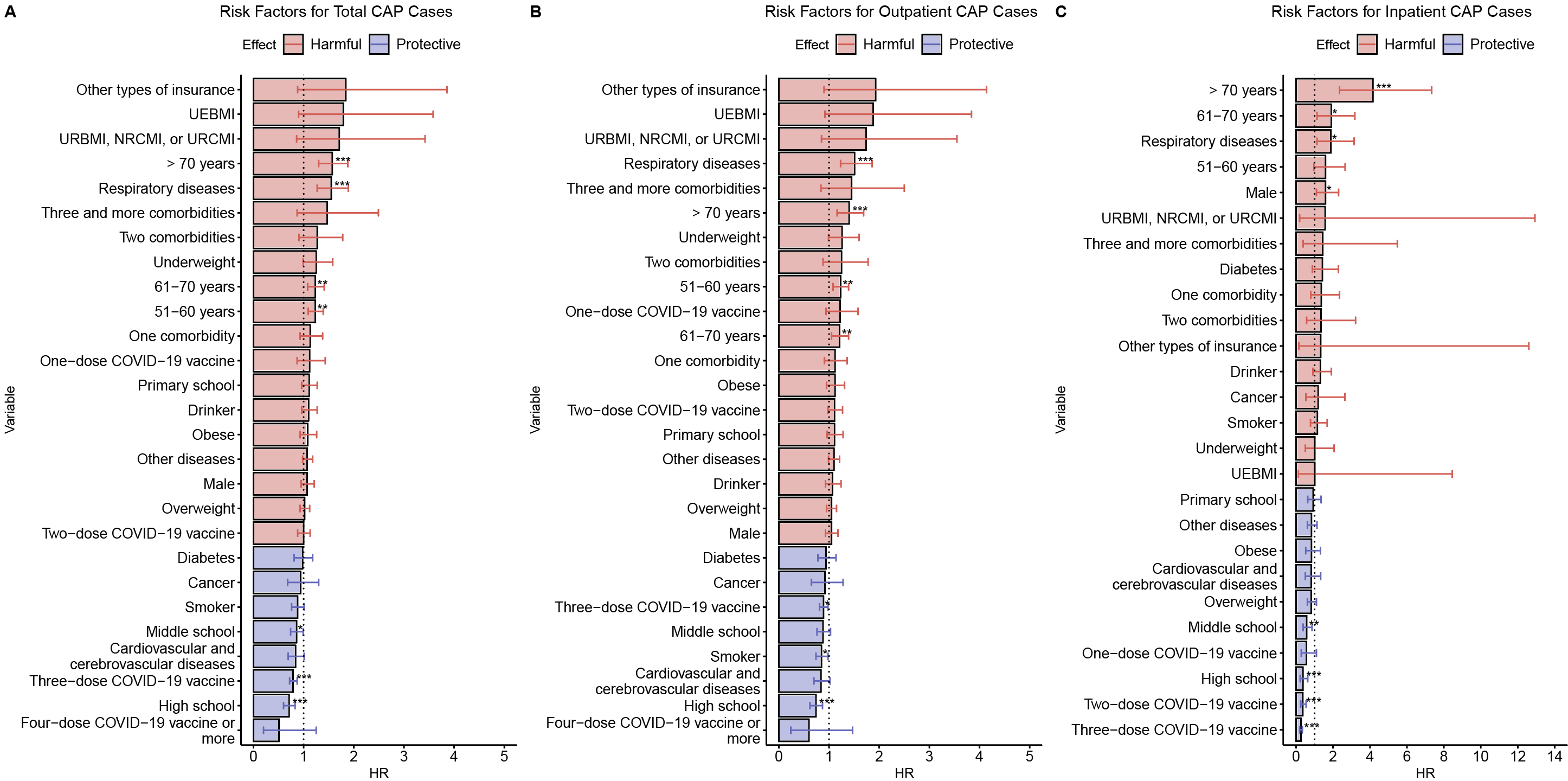


**Fig. S6 Risk factors of CAP during NPIs using multivariate models.**


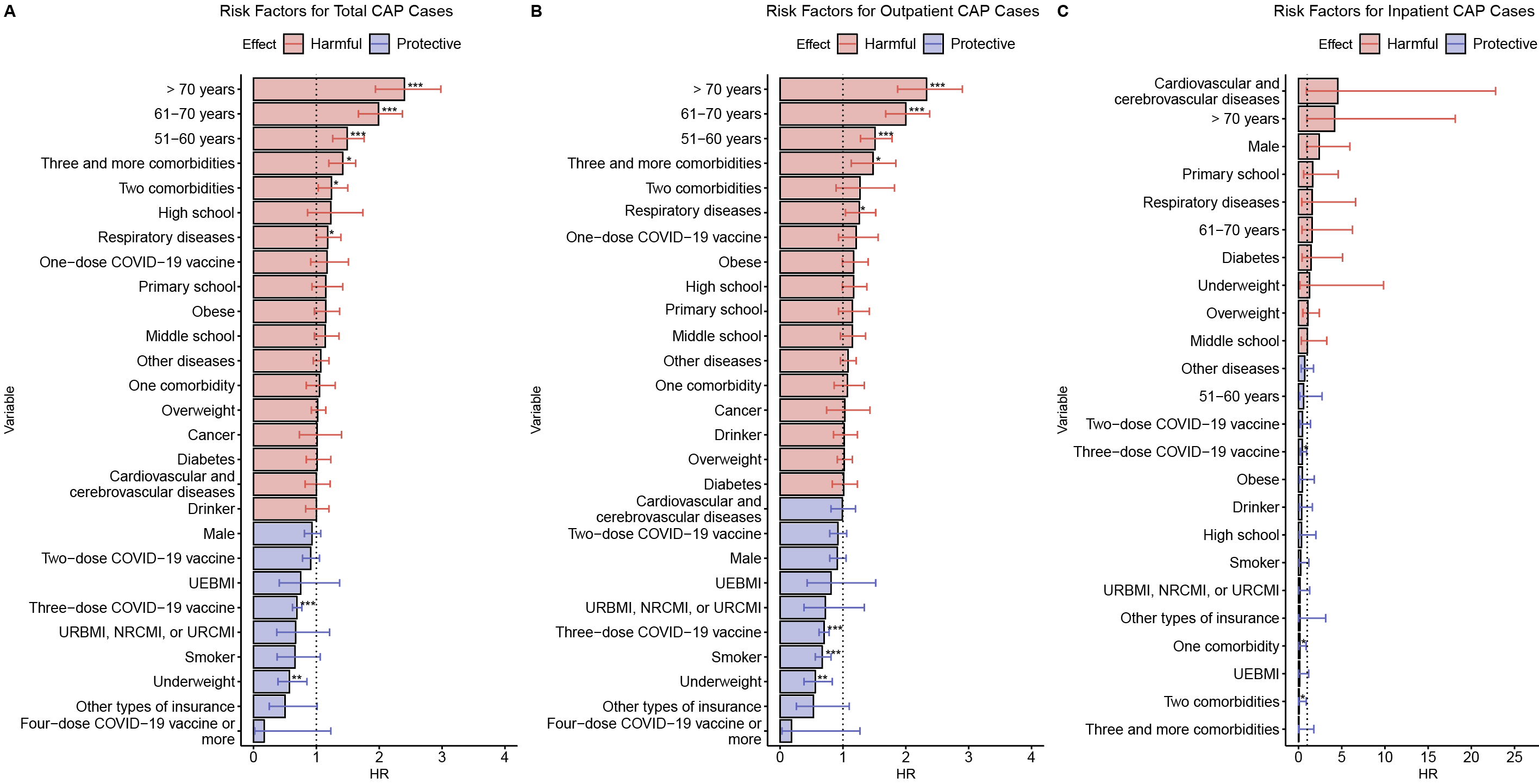
 **Fig. S7 Risk factors of CAP after NPIs using multivariate models.**
