# Supplementary figures and images for: Incidence of community-acquired pneumonia among adults between 2016 and 2023: an observational cohort study
Source: Epidemiol Infect. 2026 Jan 6;154:e15. doi: 10.1017/S0950268825100897 (PMC12835933; doi:10.1017/S0950268825100897)

a)

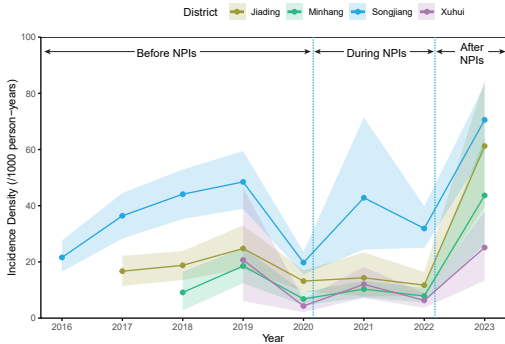

b)

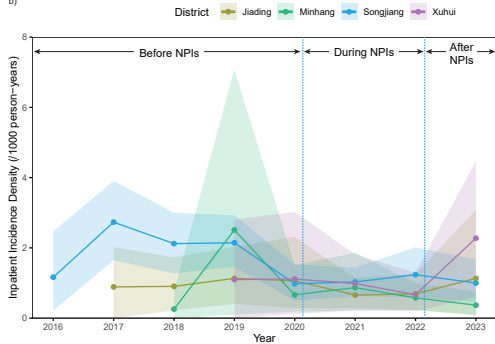

c)

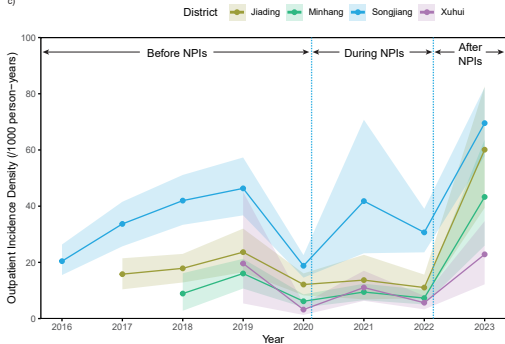

d)

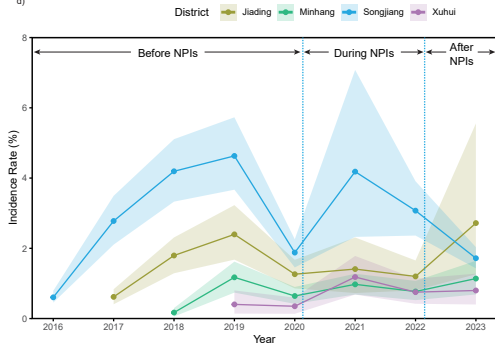

Supplement: Wang et al. supplementary material [file S0950268825100897sup001.zip › Figure S1-1016.pdf]
